# Supplementary material for: The IMpact of Vertical HIV infection on child and Adolescent SKeletal development in Harare, Zimbabwe (IMVASK Study): a protocol for a prospective cohort study
Source: BMJ Open. 2020 Feb 9;10(2):e031792. doi: 10.1136/bmjopen-2019-031792 (PMC7045196; doi:10.1136/bmjopen-2019-031792)

BMJ Open

BMJ Open is committed to open peer review. As part of this commitment we make the peer review history of every article we publish publicly available.

When an article is published we post the peer reviewers' comments and the authors' responses online. We also post the versions of the paper that were used during peer review. These are the versions that the peer review comments apply to.

The versions of the paper that follow are the versions that were submitted during the peer review process. They are not the versions of record or the final published versions. They should not be cited or distributed as the published version of this manuscript.

BMJ Open is an open access journal and the full, final, typeset and author-corrected version of record of the manuscript is available on our site with no access controls, subscription charges or pay-per-view fees (http://bmjopen.bmj.com).

If you have any questions on BMJ Open's open peer review process please email info.bmjopen@bmj.com

BMJ Open

The IMpact of Vertical HIV infection on child and Adolescent Skeletal development in Harare, Zimbabwe (IMVASK Study): a protocol for a prospective cohort study

Journal:	*BMJ Open*
Manuscript ID	bmjopen-2019-031792
Article Type:	Protocol
Date Submitted by the Author:	18-May-2019
Complete List of Authors:	Rukuni, Ruramayi; London School of Hygiene and Tropical Medicine, Clinical Research Department; Biomedical Research and Training Institute, Harare<br>Gregson, Celia; University of Bristol, Musculoskeletal Research Unit; Royal United Hospital NHS Trust, Older Person's Unit<br>Kahari, Cynthia; London School of Hygiene and Tropical Medicine, 4. Department of Infectious Disease Epidemiology, Faculty of Epidemiology and Population Health; Biomedical Research and Training Institute (BRTI)<br>Kowo, Farirayi; University of Zimbabwe, Department of Radiology<br>McHugh, Grace; Biomedical Research and Training Institute, Harare<br>Munyati, Shungu; Biomedical Research and Training Institute, Harare<br>Mujuru, Hilda; University of Zimbabwe , College of Health Sciences<br>Ward, Kate; MRC Lifecourse Epidemiology Unit<br>Filteau, Suzanne; London School of Hygiene & Tropical Medicine, Population Health<br>Rehman, Andrea; London School of Hygiene and Tropical Medicine, Infectious Disease Epidemiology<br>Ferrand, Rashida; London School of Hygiene and Tropical Medicine
Keywords:	HIV & AIDS < INFECTIOUS DISEASES, Tropical medicine < INFECTIOUS DISEASES, Paediatric radiology < PAEDIATRICS, Epidemiology < TROPICAL MEDICINE

SCHOLARONE™
Manuscripts

I, the Submitting Author has the right to grant and does grant on behalf of all authors of the Work (as defined in the below author licence), an exclusive licence and/or a non-exclusive licence for contributions from authors who are: i) UK Crown employees; ii) where BMJ has agreed a CC-BY licence shall apply, and/or iii) in accordance with the terms applicable for US Federal Government officers or employees acting as part of their official duties; on a worldwide, perpetual, irrevocable, royalty-free basis to BMJ Publishing Group Ltd ("BMJ") its licensees and where the relevant Journal is co-owned by BMJ to the co-owners of the Journal, to publish the Work in this journal and any other BMJ products and to exploit all rights, as set out in our licence.

The Submitting Author accepts and understands that any supply made under these terms is made by BMJ to the Submitting Author unless you are acting as an employee on behalf of your employer or a postgraduate student of an affiliated institution which is paying any applicable article publishing charge ("APC") for Open Access articles. Where the Submitting Author wishes to make the Work available on an Open Access basis (and intends to pay the relevant APC), the terms of reuse of such Open Access shall be governed by a Creative Commons licence – details of these licences and which Creative Commons licence will apply to this Work are set out in our licence referred to above.

Other than as permitted in any relevant BMJ Author's Self Archiving Policies, I confirm this Work has not been accepted for publication elsewhere, is not being considered for publication elsewhere and does not duplicate material already published. I confirm all authors consent to publication of this Work and authorise the granting of this licence.

For peer review only

The IMpact of Vertical HIV infection on child and Adolescent Skeletal development in Harare, Zimbabwe (IMVASK Study): a protocol for a prospective cohort study

Ruramayi Rukuni[1,2], Celia L Gregson[3], Cynthia Kahari[2,4], Farirayi Kowo[5],Grace McHugh[2], Shungu Munyati[2], Hilda Mujuru[6], Kate A Ward[7], Suzanne Filteau[8], Andrea M Rehman[4] and Rashida A Ferrand[1,2]

Affiliations:

1. Clinical Research Department, Faculty of Infectious and Tropical Diseases, London School of Hygiene and Tropical Medicine (LSHTM), London, UK.
2. Biomedical Research and Training Institute (BRTI), Harare, Zimbabwe.
3. The Musculoskeletal Research Unit, Translational Health Sciences, Bristol Medical School, University of Bristol, Bristol, UK.
4. Department of Infectious Disease Epidemiology, Faculty of Epidemiology and Population Health, London School of Hygiene and Tropical Medicine (LSHTM), London, UK.
5. Department of Radiology, University of Zimbabwe, Harare, Zimbabwe.
6. Department of Paediatrics, University of Zimbabwe, Harare, Zimbabwe.
7. MRC Lifecourse Epidemiology Unit, University of Southampton, Southampton, UK.
8. Department of Population Health, Faculty of Epidemiology and Population Health, London School of Hygiene and Tropical Medicine (LSHTM), London, UK.

Corresponding Author

Dr Ruramayi Rukuni

Biomedical Research and Training Institute (BRTI), 10 Seagrave Rd, Avondale, Harare, Zimbabwe.

Tel: +263 719 362 961

Email: Ruramayi.Rukuni@lshtm.ac.uk

For peer review only - http://bmjopen.bmj.com/site/about/guidelines.xhtml

ABSTRACT

Introduction

The scale-up of antiretroviral therapy (ART) across sub-Saharan Africa (SSA) has reduced mortality such that increasing numbers of children with perinatally acquired HIV infection are surviving to adolescence. However, children with HIV (CWH) experience a range of morbidities due to chronic HIV infection and its treatment. Impaired linear growth (stunting), is a common manifestation, affecting up to 50% of children. However, the effect of HIV on bone and muscle development during adolescent growth is not well characterised. Given the close link between pubertal timing and musculoskeletal development, any impairments in adolescence are likely to impact on future adult musculoskeletal health. We hypothesize that bone and muscle mass accrual in CWH is reduced, putting them at risk of reduced bone mineral density (BMD) and muscle function and increasing fracture risk. This study aims to determine the impact of HIV on BMD and muscle function in peri-pubertal children on ART in Zimbabwe.

Methods and analysis

CWH (n=300) aged 8-16 years established on ART, and children without HIV (n=300) frequency matched for age and sex will be recruited into a prospective cohort study. Musculoskeletal assessments including dual-energy X-ray absorptiometry (DXA), peripheral quantitative computed tomography (pQCT), grip strength and standing long jump will be conducted at baseline and after one year. The primary study outcomes are size-adjusted mean bone density Z-scores (*i.e.* total-body less-head (TBLH) bone mineral content (BMC) for lean mass adjusted for height (TBLH BMC^{LBM}) and lumbar spine bone mineral apparent density (LS BMAD) Z-scores by HIV status and the baseline prevalence of low size-adjusted BMD (i.e. Z-scores <-2) by HIV status.

Ethics and dissemination

Ethical approval for this study has been granted by the Medical Research Council of Zimbabwe and the LSHTM Ethics Committee. Baseline and longitudinal analyses will be published in peer reviewed journals and disseminated to research communities.

For peer review only - http://bmjopen.bmj.com/site/about/guidelines.xhtml

STRENGTHS AND LIMITATIONS OF THIS STUDY

- This study will provide novel understanding of the effects of HIV on bone and muscle development in a large population of sub-Saharan African (SSA) children living with HIV at the critical period of pubertal growth by using 'gold standard' size adjustment methods for DXA, which are crucial for assessing a population with inherent size differences
- Most publications of bone health in perinatal HIV to date have been cross-sectional. This prospective study will provide understanding of bone and muscle changes over time
- Bone architecture measurement by pQCT will provide understanding of trabecular and cortical bone geometry and strength in CWH
- This study will generate new data for total body and lumbar spine DXA, tibial pQCT, hand grip strength and standing long jump for Zimbabwean children without HIV which will inform normative reference data
- Whilst the age range in this study, 8-16 years, will allow analysis of pubertal delay in children with HIV, the follow-up period is insufficient to determine the impact on attainment of peak bone mass

For peer review only

INTRODUCTION

Sub-Saharan Africa (SSA) disproportionally bears the burden of global HIV infection, with nearly 90% of the estimated 2.1 million children under 15 years of age living in SSA [1]. The global scale-up of ART has dramatically improved survival of children with HIV (CWH) [2]. However there is accumulating evidence that the growing number of these children are now reaching adolescence in SSA with multisystem chronic comorbidities associated with HIV infection and/or its treatment [3].

Poor linear growth (*i.e*. stunting), is one of the most common manifestations of perinatally-acquired HIV infection, affecting up to 50% of children [4]. Linear growth is greatest in adolescence during the pubertal development period. Approximately 40% of peak bone mass (PBM), the maximum amount of bone accrued by the end of skeletal maturation, is attained during adolescent growth (Figure 1) [5]. After PBM is reached, there is no net gain in bone mass. Therefore PBM is the net reservoir of bone for later life, a key determinant of adult BMD and consequently of adult osteoporotic fracture risk [6-8]. Linear growth is therefore intimately linked to skeletal development but how HIV infection affects bone development in peri-pubertal SSA children is largely unknown. The prevalence of low BMD has been found to be higher in CWH than uninfected children in high and middle income countries (7% in the USA [9], 32% in Brazil [10] and 24% in Thailand [11] compared to 1% in children without HIV in the

For peer review only - http://bmjopen.bmj.com/site/about/guidelines.xhtml

USA [9]. No study has estimated the prevalence of low BMD in SSA, and the prevalence of and risk factors for low BMD in African CWH are not known [12, 13]. It is important to highlight that the risk of poor bone accrual, reflected in low BMD measurements, is likely to be different in low income countries compared to high income countries due to factors such as malnutrition and social deprivation; but critically due to delayed ART initiation. A recent meta-analysis has shown that the median age of ART initiation in the UK/USA is two years, compared to eight years in SSA [14].

The mechanisms by which HIV may lead to low size-adjusted BMD in children are not fully understood but are likely multifactorial including HIV-associated factors (*e.g.* ART drugs, HIV disease stage) and traditional risk factors (*e.g.* hypogonadism, smoking, alcohol, low physical activity and vitamin D deficiency) [15]. HIV infection promotes systemic immune activation and production of inflammatory cytokines (*e.g.* TNFα) that in turn promote increased bone resorption [16]. ART initiation, particularly with tenofovir (part of the first-line ART regimen in SSA), predicts an initial decline in BMD which stabilizes after two years in adults [17]. It is thought tenofovir may cause renal proximal tubule toxicity resulting in phosphate wasting and increased bone turnover [18]. Although tenofovir and protease inhibitors have been associated with low bone mineral density (BMD) in adults [19, 20], studies in children have shown inconsistent findings [21-23]. Malnutrition, opportunistic infections and social deprivation may also impede musculoskeletal development. Reduced physical activity, associated with HIV [24], may also impair muscle development and limit impact loading to reduce osteocyte-mediated bone accrual [25, 26] . In adults, weak grip strength has been associated with increased falls and fracture risk [27]. Although lean muscle mass has also been shown to predict the magnitude of bone accrual during growth [28], few studies have compared muscle strength and function between children with and without HIV. Interestingly, a small Canadian study showed deficits in muscle power in CWH [29].

Another mechanism by which HIV may exert effects on BMD is through its effect on puberty. Even in the presence of ART, the onset of puberty is delayed by approximately a year in CWH in both high income [30] and low income settings [31]. Pubertal delay in HIV may be mediated through nutritional deficiency, recurrent infection, or chronic immune activation disrupting hormonal regulation [31]. Delayed puberty may be advantageous for linear growth; spending more time in puberty may allow more time for skeletal growth [31]. Conversely, delayed puberty has been shown in studies in high income settings to be detrimental to bone mass accrual [32, 33]. However, the impact of pubertal delay on BMD in low income countries remains unknown. Pubertal delay can be assessed objectively using hand radiographs. Analysis of the growth plate development and fusion of long bones in the

For peer review only - http://bmjopen.bmj.com/site/about/guidelines.xhtml

hands can accurately quantify bone age, which is a measure of skeletal maturation. Bone age lagging behind chronological age reflects pubertal delay [34].

BMD is commonly measured by DXA as two-dimensional (areal) BMD, however, this is highly dependent on bone size [35]. DXA underestimates areal bone density in short children, with smaller bones, and overestimates BMD in taller children, with bigger bones, despite the fact that they may have identical volumetric BMD. Size adjustment of DXA measures is therefore critically important in children with chronic diseases such as HIV, where smaller size due to poorer growth and delayed puberty may explain findings of lower BMD. The two 'gold standard' size-adjustment techniques chosen from the International Society for Clinical Densitometry (ISCD) are: bone mineral apparent density at the lumbar spine (LS BMAD) and regression based total-body less-head (TBLH) Bone Mineral Content (BMC) for lean mass adjusted for height (TBLH BMC^{LBM}) [36] Z-scores. As there are currently no published reference DXA data for child or adolescent populations in SSA, in this study we will use the of best available data sets from high income countries such as the UK [36] to generate Z-scores.

Unlike DXA, peripheral quantitative computed tomography (pQCT) takes into account bone size by directly measuring volumetric BMD. It has the additional advantage of separately assessing trabecular and cortical bone compartments, providing information on bone architecture. Furthermore, a range of bone strength indices *e.g.* strength stain index, validated against fracture risk can be calculated [37, 38]. In high income countries, markedly abnormal trabecular and cortical architecture have been shown in adults with HIV [39] and abnormal bone architecture and impaired bone strength through to early adulthood have been shown in boys with HIV infection [39]. Few studies have assessed bone architecture and strength in CWH in SSA.

The IMVASK study aims to determine the prevalence of low size-adjusted BMD and muscle function (grip strength and standing long jump) in Zimbabwean children with and without HIV. pQCT assessment will enable understanding of the impact of HIV infection on bone architecture and strength. This study will further contribute to local reference data for DXA measures, bone age and muscle function (grip strength and standing long jump) for a sub-Saharan African population, establishing a biorepository for future research. Study results will aid understanding of bone and muscle accrual in the context of HIV infection in the era of ART.

For peer review only - http://bmjopen.bmj.com/site/about/guidelines.xhtml

METHODS AND ANALYSIS

Study objectives

To determine the impact of HIV infection on size-adjusted bone density in peri-pubertal children aged 8-16 years established on antiretroviral therapy (ART). The objectives of this prospective study are:

1) To quantify the prevalence of low size-adjusted BMD and low muscle function (grip strength and standing long jump) among HIV-infected children compared to uninfected children
2) To investigate the risk factors for low size-adjusted bone density and low muscle function (grip strength and standing long jump) among children with HIV
3) To compare the rates of bone mass accrual over one year between children with and without HIV
4) To determine the differences in bone architecture measured by pQCT between children with and without HIV

Study hypothesis

We hypothesize that HIV infection adversely affects skeletal development, such that children living with HIV, despite ART, accrue less bone mass and strength and have reduced muscle function during skeletal development.

Study design

CWH aged 8-16 years and established on ART (n=300) and a comparison group of children without HIV, frequency-matched for age and sex (n=300) will be recruited into a prospective cohort study. Detailed musculoskeletal assessments will be conducted at baseline and after one year.

Study setting

Parirenyatwa and Harare Hospital are the largest public-sector referral hospitals in Harare [40, 41]. The paediatric HIV clinics at both hospitals provide HIV care to more than 2,000 children. Although HIV care is increasingly decentralised to primary care level across the country, most children in Harare continue to receive care within HIV clinics in secondary healthcare facilities. Parirenyatwa hospital has a well-functioning radiology department which houses the University of Zimbabwe DXA and pQCT research unit and has access to private radiology services in the surrounding area. The hospital catchment areas have over 116 primary and 42 secondary government schools with an estimated 157,962 children enrolled [42]. School attendance in Harare province is high and does not differ by HIV status, with 96% of children under 18 years attending school [43, 44].

For peer review only - http://bmjopen.bmj.com/site/about/guidelines.xhtml

Recruitment of participants

Eligibility

Inclusion criteria: age 8-16 years (includes pre- and peri-pubertal children), living in Harare, and in CWH only if:

i. taking ART for at least two years (as adult studies demonstrate ART initiation is followed by an initial decline in BMD which stabilizes after 2 years [17]).

ii. the child is aware of their HIV status, to avoid inadvertent disclosure as a result of study participation.

Exclusion criteria: acute illness (requiring immediate hospitalization) and lack of consent.

Recruitment of children with HIV

Systematic quota-based sampling by age and sex will be used to recruit 300 children from Parirenyatwa and Harare Hospital HIV clinics. Participants will be recruited sequentially as they attend clinic such that 50 males and 50 females will be chosen for each of three age-strata, 8-10.99, 11-13.99 and 14-16.99 years. A maximum of 5 participants will be enrolled on each day for logistical reasons. The total number of children approached each day will be recorded, irrespective of whether they are subsequently eligible or enrolled to determine the sampling fraction.

Recruitment of children without HIV

Three hundred HIV-uninfected children will be randomly sampled from six government primary and secondary schools in the same catchment area as Parirenyatwa and Harare Hospitals. Younger children (8-12 years) will be selected from primary schools and older children (13-16 years) from secondary schools, with thirteen-year olds coming from both primary and secondary schools. The number of children selected from each school will be proportional to school size, thereby giving each child equal probability of being sampled. A random number sequence will be generated, and school registers will be used to select participants of similar age and sex as the children with HIV using the same quota-based approach of 50 males and 50 females in each of the three age strata. Guardians of selected school children will be invited to the study clinic to complete the consent process. Consenting participants will have a diagnostic HIV test as part of their assessment. Those testing HIV positive (anticipated to be approximately 2-3% [45]) will be referred for HIV care.

Study procedures

Questionnaire

An interviewer-administered questionnaire together with hand-held medical records will be used to collect socio-demographic details and clinical history including age, sex, school attendance, orphan

For peer review only - http://bmjopen.bmj.com/site/about/guidelines.xhtml

For peer review only

status, guardianship, history of fractures with mechanism of trauma, steroid use, smoking, alcohol, recreational drugs, family history of musculoskeletal disease, co-morbidities, physical activity, diet and nutrition and sun exposure. Where possible, validated instruments adapted for the local context will be used. For example, the International Physical Activity Questionnaire (IPAQ) [46] validated in multiple countries including South Africa and will be used to assess physical activity as multiples of the resting metabolic rate (MET) in MET-minutes. Diet and nutrition will be assessed using a tool we developed for the Zimbabwean context based on a validated dietary diversity and food frequency tool from India and Malawi [47] and international guidelines applicable to SSA [48]. The tool quantifies vitamin D supplementation and sunlight exposure and has been adapted to reflect the Zimbabwean context where fortification of oils and margarine with vitamin D is mandated by the government and specific vitamin D rich foods such as kapenta fish are found.

Clinical examination

A standardised musculoskeletal examination will be conducted using the validated paediatric gait, arms, legs and spine (pGALS) examination [49]. Additional clinical assessments will be carried out using standardised protocols and calibrated equipment. Anthropometry measurements will include standing and sitting height, arm span, mid upper arm circumference. Height will be measured to the nearest 0.1 cm, by two separate readers using calibrated Seca 213 stadiometers. If the two height measurements differ by more than 0.5 cm , a third reading will be taken [50]. Weight will be measured to the nearest 0.1 kg using calibrated Seca 875 scales. Tanner pubertal staging will be carried out using a standardised protocol with an orchidometer to assess testicular volume in males [51, 52]. Muscle function will be assessed in the upper limb and lower limbs by grip strength dynamometry and standing long jump respectively. Hand grip strength will be measured using a Jamar hydraulic hand-held dynamometer (Patterson Medical, UK) to the nearest 0.1kg. Participants will be seated with the shoulder at 0° to 10°, the elbow at 90° of flexion and the forearm positioned neutrally. Three measurements will be taken from each hand in alternation and the highest measurement chosen. The standing long jump distance will be taken from the best of three correctly performed attempts to the nearest 0.1 cm, measuring the distance from the take-off line to the heel.

Radiological assessments

DXA scans will be performed by two trained radiographers using a Hologic QDR Wi densitometer with Apex software version 4.5. Measurements will be taken from the lumbar spine, hip and total body. Fat and muscle mass will also be acquired; muscle mass is the fat free mass measurement from DXA. DXA scans will be repeated in a subgroup (n=20) of participants to determine reproducibility. Peripheral quantitative computed tomography (pQCT) measurements of the left tibia will be taken

For peer review only - http://bmjopen.bmj.com/site/about/guidelines.xhtml

using a Stratec XCT-2000 scanner (Stratec, Pforzheim, Germany) software version 6.20. Measurements of the left tibia will be taken at three sites at 4%, 38%, and 66% percent of the tibial length, measured from the medial malleolus to the medial tibial plateau. Daily quality control will be performed by scanning the manufacturer provided lumbar spine phantom for DXA and tibia phantom for pQCT. A radiograph of the non-dominant hand and wrist will be taken and used to quantify bone age using the Greulich and Pyle (G&P) atlas and the Tanner Whitehouse 3 (TW3) method. For Intra-observer reliability, 10% of the radiographs will be randomly selected and rescored by the same operator after one week. For inter-observer reliability a different set of 10% of the radiographs will be re-scored by a different expert. The estimated bone age will then be compared to the calculated chronological age.

Blood tests

A fasting blood sample (up to 15ml) will be collected at enrolment. HIV markers (CD4 count and viral load) will be tested in CWH only. CD4 cell count will be measured using an Alere PIMA CD4 machine (Waltham, Massachusetts, USA). HIV viral load will be measured using the GeneXpert HIV-1 viral load platform (Cepheid Inc, Sunnyvale, California, USA). The remaining blood plasma will be bio-banked to enable future measurement of bone biochemistry. After removing the plasma, peripheral blood mononuclear cells (PBMC) will be isolated and cryopreserved. DNA will also be extracted using a manual method and stored for future genetic studies.

Follow up at one year

All study measurements, with the exception of DNA extraction, will be repeated after one year. Participants will be recalled exactly one year after their first DXA scan. The aim is to perform all scans within a 4-week window period. Contact will be maintained with participants via regular phone calls and text messaging to minimise loss-to-follow-up. The schedule of study procedures is summarised in Table 1.

Outcome measures

The primary study outcomes are:

1) mean size-adjusted bone density Z-scores; TBLH BMC^{LBM} and LS BMAD [36] by HIV status.
2) the prevalence of low TBLH BMC^{LBM} and LS BMAD Z-score <-2 at baseline, by HIV status [36].

Secondary study outcomes are:

1) prevalence of low muscle function; grip strength and standing long jump-for-age (Z-score<-2) and musculoskeletal abnormalities/disabilities by HIV status at baseline.

For peer review only - http://bmjopen.bmj.com/site/about/guidelines.xhtml

2) mean percentage change in TBLH BMC^{LBM} (g) and LS BMAD (g/cm^3), tibial cortical and trabecular volumetric BMD (g/cm^3), total cross sectional area, cortical thickness and bone strength, muscle mass and function at baseline and one year, by HIV status.
3) assessment of the extent to which pubertal delay explains changes in these bone and muscle outcomes.

Sample size

The sample size was calculated to detect differences in DXA-measured mean size-adjusted bone BMD Z-scores between children with and without HIV. This study will have 80% power (α 0.05) to detect a 0.23 Z-score difference between 300 HIV-infected and 300 uninfected children, assuming a standard deviation of 1.3. As there were no published studies from low income countries, estimates of the expected difference were taken from a US study of children with HIV aged 7 to 15 years [9]. In addition, our study will have 80% power to detect a 4.8% difference in the prevalence low size-adjusted BMD between the two groups. This is a smaller prevalence difference than that detected by the most conservative prevalence estimate of low BMD of 7% from three studies in high- and middle-income countries [9-11].

Statistical analysis

Baseline mean TBLH BMC^{LBM} and LS BMAD Z-scores and the prevalence of low TBLH BMC^{LBM} and LS BMAD Z-score by HIV status at baseline will be determined. Among CWH, the association between and *a priori* defined risk factors (ART duration, ART type, proportion of life on treatment, age at ART initiation, CD4 count, viral load, bone age, pubertal stage, nutrition, socioeconomic status and orphanhood) against size-adjusted BMD will be examined using multiple linear regression (Z-score as a continuous variable) and multivariate logistic regression (as defined by the Z-score cut off of <-2).

Multiple linear regression will be used to analyse the mean difference in % change in TBLH BMC^{LBM}(g) and LS BMAD (g/cm^3) between children with and without HIV. Models will be adjusted for physical activity and calcium and vitamin D intake. Interaction between the effects of pubertal stage (bone age) and HIV on change in TBLH BMC^{LBM} and LS BMAD will be investigated to see if differences in bone density become more or less pronounced through puberty *i.e*. whether catch-up growth is possible, see Figure 2. If the regression coefficient (β) is markedly more positive in CWH, this will suggest that catch-up growth is possible as shown by Figure 2D. Data for total body and lumbar spine DXA, tibial pQCT, hand grip strength and standing long jump in CWH will be analysed with reference to the comparator group of children without HIV.

For peer review only - http://bmjopen.bmj.com/site/about/guidelines.xhtml

For the purposes of normative data derivation, children without HIV who have any diagnosis or evidence of muscle or bone disease will be excluded. Then outliers with bone density, hand grip strength or standing long jump data beyond 2 standard deviations from the mean will have their case record reviewed to exclude cases with underlying bone or muscle pathology. The remaining population will be used to generate normative references ranges for these quantitative traits.

Data management

Data collection, management and storage will be governed by standard operating procedures and will follow the principles of Good Clinical Practice (GCP). Data will be captured using hand held tablets for the questionnaires. Paper forms will be available in case of failure of electronic data entry. Microsoft Access will be used as the main backend database as it allows programming of quality control checks and conditional data validation. GCP compliant audit trail modules will be incorporated into the databases and reports of aggregated data will be reviewed on a monthly basis. In order to assure data quality and consistency, all staff will receive regular training and regular quality checks will be conducted. Paper records will be stored for eight years after the completion of research in secure, locked storage facilities. Field staff will download data to the central database, which is backed up onto an encrypted external hard drive daily, and to additional off-site and secure cloud back-up. The off-site back-up copies will be stored through the London School of Hygiene and Tropical Medicine (LSHTM) Research Data Management Support Service that has an established data repository. In order to preserve the long-term value of this data, it will be stored backed-up here indefinitely. Anonymised research data will be made available for sharing through the open access data repository established by the LSHTM Data Management Support Service at the time of publication. This will allow other research groups to request access to study data and tools. Information on how other researchers' data will be included in every study publication.

Patient and public involvement

As part of public involvement, there are plans to carry out science fairs at the secondary schools where children without HIV are recruited from. The aim of these fairs is to engage young people, their parents/guardians and community about the value of science and health research through their schools. We hope to inspire students about science and create opportunities to interact with health care professionals to ignite career aspirations using musculoskeletal health research as a model. We will provide support for teachers who deliver school science curricula by providing exiting learning opportunities outside the traditional classroom environment.

For peer review only - http://bmjopen.bmj.com/site/about/guidelines.xhtml

Additional funding has been secured to run a series of science fairs at the three secondary schools over one year. The science fairs will foster a creative environment through interactive games and the use of audio-visual media and a supporting website. Students will also be invited to present their own science projects and to submit entries to a short essay prize competition about how science can be used to solve a local problem in their community. This format for the science fairs has been successfully piloted at one school and will be rolled out to the other schools.

Study status

Recruitment to this study began in May 2018 and is planned until May 2019. Study follow up will run from May 2019 to May 2020.

For peer review only

DISCUSSION

Although the scale-up of prevention of mother-to-child transmission (PMTCT) has reduced perinatal HIV transmission but coverage is still not universal in most parts of SSA and therefore perinatal HIV infection is expected to affect large numbers of children for years to come. Furthermore, the scale-up of ART has reduced HIV-associated mortality dramatically so that CWH, who would previously have died in infancy or early childhood, are now reaching adolescence in increasing numbers. It is therefore important to understand the impact of HIV infection and its treatment on skeletal development during the critical period of puberty.

This study will determine the prevalence of low size-adjusted BMD in children with and without HIV in Zimbabwe, a country with a severe sustained early onset HIV epidemic. In addition, this study will determine risk factors for low size-adjusted BMD in CWH. We aim to identify factors amenable to intervention, which may be modifiable to maximize future bone health and minimize subsequent adult osteoporotic fracture risk. For example, reduced muscle function predicting low size-adjusted BMD, may suggest targeted physiotherapy would be of benefit which would warrant formal investigation.

Our study will provide insights regarding the mechanisms through which perinatal HIV infection affects the timing of pubertal onset and bone mass accrual. By measuring bone and muscle parameters at baseline and one year and employing 'gold standard' size-adjustment methodology for DXA-measured BMD in the growing skeleton, this study will also provide insights into whether catch-up growth in terms of bone mass accrual is possible in HIV despite pubertal delay.

The bone architecture measured by pQCT in this study will provide separate assessments of trabecular and cortical bone density, and bone geometry and strength in Zimbabwean children. The evidence

For peer review only - http://bmjopen.bmj.com/site/about/guidelines.xhtml

from studies in adult men established on ART demonstrate impairments in trabecular and cortical bone architecture [53]. Whether the same applies to children needs to be determined.

Furthermore, we will establish novel comparator data for DXA, pQCT, bone age, hand grip strength and standing long jump for a Zimbabwean population, which will be able to be used for future research in this context. This study will establish a biorepository for future research *e.g.* potential bone turnover marker measurement and genotyping.

Given the magnitude of the HIV epidemic in SSA and the large cohort of young people who may experience impaired bone accrual, musculoskeletal disability or fracture as they reach adolescence and early adulthood; it is imperative to characterise the impact of perinatal HIV on musculoskeletal development.

ETHICS AND DISSEMINATION

Ethical approval has been granted by the London School of Hygiene and Tropical Medicine Ethics Committee (Ref: 15333; 14 May 2018), the Institutional Review Board of the Biomedical Research and Training Institute (Ref: AP 145/2018; 20 February 2018), the Joint Research Ethics Committee for University of Zimbabwe College of Health Sciences and the Parirenyatwa Group of Hospitals (JREC) (Ref: 11/18; 1 March 2018), Harare Central Hospital Ethics Committee (HCHEC) (Ref: 170118/04; 23 February 2018), the Medical Research Council of Zimbabwe Ref: (MRCZ/A/2297; 10 April 2018) and the Ministry of Primary and Secondary Education Zimbabwe (Ref: C/426/Harare; 13 February 2018).

Study progress will be reported annually to MRCZ. Results of interim data analysis will be presented at national and international research meetings and conferences. Study findings will be published in international peer reviewed scientific journals and disseminated to research communities at the end of study.

AUTHORS' CONTRIBUTIONS

RR, RF and CG co-designed the study. RR wrote the study protocol and was responsible for journal selection and preparation of the first draft of this article as the principal author. CK contributed to the development of the pQCT protocols. FK contributed to the development of the bone age analysis protocols. KW provided scan protocols, contributed to the study design, and gave methodological input regarding bone density size-adjustment and analysis. AR contributed to the study design, in particular, sampling strategy, sample size calculation and the statistical analysis plan. SF provided advice regarding the development of nutritional assessment tools. GM, HM and SM advised on study

For peer review only - http://bmjopen.bmj.com/site/about/guidelines.xhtml

conduct and provided study oversight. All authors reviewed and provided feedback on the manuscript prior to submission.

FUNDING STATEMENT

This study is funded by the Wellcome Trust UK. RR is funded by Wellcome Trust UK grant number 206764/Z/17/Z. CK is funded by a NIH Fogharty Fellowship. RAF is funded by Wellcome Trust grant number 206316/Z/17/Z. Global challenges research funding from the University of Bristol established the Sub-Saharan African MuSculOskeletal Network (SAMSON) enabling the provision of pQCT in Zimbabwe for this study https://thesamson.org [54].

COMPETING INTERESTS STATEMENT

The authors have no competing interests to declare.

For peer review only

For peer review only - http://bmjopen.bmj.com/site/about/guidelines.xhtml

REFERENCES

1. **Monitoring the Situation of Children and Women; Global and regional trends, current status and progress.** [https://data.unicef.org/topic/hivaids/global-regional-trends/#]
2. Celletti F, Sherman G Fau - Mazanderani AH, Mazanderani AH: **Early infant diagnosis of HIV: review of current and innovative practices**. (1746-6318 (Electronic)).
3. Lowenthal ED, Bakeera-Kitaka S, Marukutira T, Chapman J, Goldrath K, Ferrand RA: **Perinatally acquired HIV infection in adolescents from sub-Saharan Africa: a review of emerging challenges**. *Lancet Infect Dis* 2014, **14**(7):627-639.
4. Arpadi SM: **Growth failure in children with HIV infection**. *J Acquir Immune Defic Syndr* 2000, **25 Suppl 1**:S37-42.
5. Bailey DA, McKay Ha Fau - Mirwald RL, Mirwald Rl Fau - Crocker PR, Crocker Pr Fau - Faulkner RA, Faulkner RA: **A six-year longitudinal study of the relationship of physical activity to bone mineral accrual in growing children: the university of Saskatchewan bone mineral accrual study**. 1999(0884-0431 (Print)).
6. Hui SL, Slemenda CW, Johnston CC, Jr.: **The contribution of bone loss to postmenopausal osteoporosis**. *Osteoporos Int* 1990, **1**(1):30-34.
7. Marshall D, Johnell O, Wedel H: **Meta-analysis of how well measures of bone mineral density predict occurrence of osteoporotic fractures**. *BMJ* 1996, **312**(7041):1254-1259.
8. Hernandez CJ, Beaupré GS, Carter DR: **A theoretical analysis of the relative influences of peak BMD, age-related bone loss and menopause on the development of osteoporosis**. *Osteoporos Int* 2003, **14**(10):843-847.
9. DiMeglio LA, Wang J, Siberry GK, Miller TL, Geffner ME, Hazra R, Borkowsky W, Chen JS, Dooley L, Patel K *et al*: **Bone mineral density in children and adolescents with perinatal HIV infection**. *AIDS* 2013, **27**(2):211-220.
10. Schtscherbyna A, Pinheiro MF, Mendonca LM, Gouveia C, Luiz RR, Machado ES, Farias ML: **Factors associated with low bone mineral density in a Brazilian cohort of vertically HIV-infected adolescents**. *International Journal of Infectious Diseases* 2012, **16**(12):e872-878.
11. Puthanakit T, Saksawad R, Bunupuradah T, Wittawatmongkol O, Chuanjaroen T, Ubolyam S, Chaiwatanarat T, Nakavachara P, Maleesatharn A, Chokephaibulkit K: **Prevalence and risk factors of low bone mineral density among perinatally HIV-infected Thai adolescents receiving antiretroviral therapy**. *J Acquir Immune Defic Syndr* 2012, **61**(4):477-483.
12. Matovu FK, Wattanachanya L, Beksinska M, Pettifor JM, Ruxrungtham K: **Bone health and HIV in resource-limited settings: a scoping review**. *Curr Opin HIV AIDS* 2016, **11**(3):306-325.
13. Arpadi SM, Shiau S, Marx-Arpadi C, Yin MT: **Bone health in HIV-infected children, adolescents and young adults: a systematic review**. *J AIDS Clin Res* 2014, **5**(11).
14. Slogrove AL, Schomaker M, Davies MA, Williams P, Balkan S, Ben-Farhat J, Calles N, Chokephaibulkit K, Duff C, Eboua TF *et al*: **The epidemiology of adolescents living with perinatally acquired HIV: A cross-region global cohort analysis**. *PLoS Med* 2018, **15**(3):e1002514.
15. Casado JL, Bañon S, Andrés R, Perez-Elías MJ, Moreno A, Moreno S: **Prevalence of causes of secondary osteoporosis and contribution to lower bone mineral density in HIV-infected patients**. *Osteoporosis International* 2014, **25**(3):1071-1079.
16. Weitzmann MN: **The Role of Inflammatory Cytokines, the RANKL/OPG Axis, and the Immunoskeletal Interface in Physiological Bone Turnover and Osteoporosis**. *Scientifica (Cairo)* 2013, **2013**:125705.
17. Aurpibul L, Cressey TR, Sricharoenchai S, Wittawatmongkol O, Sirisanthana V, Phongsamart W, Sudjaritruk T, Chokephaibulkit K: **Efficacy, safety and pharmacokinetics of tenofovir disoproxil fumarate in virologic-suppressed HIV-infected children using weight-band dosing.[Erratum appears in Pediatr Infect Dis J. 2015 Aug;34(8):847]**. *Pediatric Infectious Disease Journal* 2015, **34**(4):392-397.

For peer review only - http://bmjopen.bmj.com/site/about/guidelines.xhtml

18. Fux CA, Rauch A, Simcock M, Bucher HC, Hirschel B, Opravil M, Vernazza P, Cavassini M, Bernasconi E, Elzi L *et al*: **Tenofovir use is associated with an increase in serum alkaline phosphatase in the Swiss HIV Cohort Study**. *Antivir Ther* 2008, **13**(8):1077-1082.
19. Hansen AB, Obel N, Nielsen H, Pedersen C, Gerstoft J: **Bone mineral density changes in protease inhibitor-sparing vs. nucleoside reverse transcriptase inhibitor-sparing highly active antiretroviral therapy: data from a randomized trial**. *HIV Med* 2011, **12**(3):157-165.
20. McComsey GA, Tebas P, Shane E, Yin MT, Overton ET, Huang JS, Aldrovandi GM, Cardoso SW, Santana JL, Brown TT: **Bone disease in HIV infection: a practical review and recommendations for HIV care providers**. *Clin Infect Dis* 2010, **51**(8):937-946.
21. Gafni RI, Hazra R, Reynolds JC, Maldarelli F, Tullio AN, DeCarlo E, Worrell CJ, Flaherty JF, Yale K, Kearney BP *et al*: **Tenofovir disoproxil fumarate and an optimized background regimen of antiretroviral agents as salvage therapy: impact on bone mineral density in HIV-infected children**. *Pediatrics* 2006, **118**(3):e711-718.
22. Purdy JB, Gafni RI, Reynolds JC, Zeichner S, Hazra R: **Decreased bone mineral density with off-label use of tenofovir in children and adolescents infected with human immunodeficiency virus**. *J Pediatr* 2008, **152**(4):582-584.
23. Giacomet V, Mora S, Martelli L, Merlo M, Sciannamblo M, Viganò A: **A 12-month treatment with tenofovir does not impair bone mineral accrual in HIV-infected children**. *J Acquir Immune Defic Syndr* 2005, **40**(4):448-450.
24. Vancampfort D, Stubbs B, Mugisha J: **Physical activity and HIV in sub-Saharan Africa: a systematic review of correlates and levels**. *African health sciences* 2018, **18**(2):394-406.
25. Santos L, Elliott-Sale KJ, Sale C: **Exercise and bone health across the lifespan**. *Biogerontology* 2017, **18**(6):931-946.
26. Santos WR, Santos WR, Paes PP, Ferreira-Silva IA, Santos AP, Vercese N, Machado DR, de Paula FJ, Donadi EA, Navarro AM *et al*: **Impact of Strength Training on Bone Mineral Density in Patients Infected With HIV Exhibiting Lipodystrophy**. *J Strength Cond Res* 2015, **29**(12):3466-3471.
27. Dodds RM, Syddall HE, Cooper R, Kuh D, Cooper C, Sayer AA: **Global variation in grip strength: a systematic review and meta-analysis of normative data**. *Age Ageing* 2016, **45**(2):209-216.
28. Bonjour JP, Chevalley T, Ferrari S, Rizzoli R: **The importance and relevance of peak bone mass in the prevalence of osteoporosis**. *Salud Publica Mex* 2009, **51 Suppl 1**:S5-17.
29. Macdonald E, Nettlefold L, Maan EJ, Cote H, Alimenti A: **Muscle power in children, youth and young adults who acquired HIV perinatally**. *J Musculoskelet Neuronal Interact* 2017, **17**(2):27-37.
30. Williams PL, Abzug MJ, Jacobson DL, Wang J, Van Dyke RB, Hazra R, Patel K, Dimeglio LA, McFarland EJ, Silio M *et al*: **Pubertal onset in children with perinatal HIV infection in the era of combination antiretroviral treatment**. *AIDS* 2013, **27**(12):1959-1970.
31. Szubert AJ, Musiime V, Bwakura-Dangarembizi M, Nahirya-Ntege P, Kekitiinwa A, Gibb DM, Nathoo K, Prendergast AJ, Walker AS, Team AT: **Pubertal development in HIV-infected African children on first-line antiretroviral therapy**. *AIDS (London, England)* 2015, **29**(5):609-618.
32. Kindblom JM, Lorentzon M, Norjavaara E, Hellqvist A, Nilsson S, Mellstrom D, Ohlsson C: **Pubertal timing predicts previous fractures and BMD in young adult men: the GOOD study**. *J Bone Miner Res* 2006, **21**(5):790-795.
33. Cousminer DL, Mitchell JA, Chesi A, Roy SM, Kalkwarf HJ, Lappe JM, Gilsanz V, Oberfield SE, Shepherd JA, Kelly A *et al*: **Genetically Determined Later Puberty Impacts Lowered Bone Mineral Density in Childhood and Adulthood**. *J Bone Miner Res* 2018, **33**(3):430-436.
34. Creo AL, Schwenk WF, 2nd: **Bone Age: A Handy Tool for Pediatric Providers**. *Pediatrics* 2017, **140**(6).

For peer review only - http://bmjopen.bmj.com/site/about/guidelines.xhtml

35. Crabtree N, Ward K: **Bone Densitometry: Current Status and Future Perspective**. In: *Calcium and Bone Disorders in Children and Adolescents. Volume Vol 28* 2nd, revised edition. edn. Edited by Allgrove J, Shaw NJ. Basel: Karger; 2015: pp 72-83.
36. Crabtree NJ, Shaw NJ, Bishop NJ, Adams JE, Mughal MZ, Arundel P, Fewtrell MS, Ahmed SF, Treadgold LA, Hogler W *et al*: **Amalgamated Reference Data for Size-Adjusted Bone Densitometry Measurements in 3598 Children and Young Adults-the ALPHABET Study**. *J Bone Miner Res* 2017, **32**(1):172-180.
37. Kontulainen SA, Johnston JD, Liu D, Leung C, Oxland TR, McKay HA: **Strength indices from pQCT imaging predict up to 85% of variance in bone failure properties at tibial epiphysis and diaphysis**. *J Musculoskelet Neuronal Interact* 2008, **8**(4):401-409.
38. Siu WS, Qin L, Leung KS: **pQCT bone strength index may serve as a better predictor than bone mineral density for long bone breaking strength**. *Journal of Bone and Mineral Metabolism* 2003, **21**(5):316-322.
39. Abubakar A, Holding P, Newton CR, van Baar A, van de Vijver FJ: **The role of weight for age and disease stage in poor psychomotor outcome of HIV-infected children in Kilifi, Kenya**. *Dev Med Child Neurol* 2009, **51**(12):968-973.
40. **Parirenyatwa Group of Hospitals: https://parihosp.org**
41. Harare Central Hospital: http://www.hararehospital.gov.zw. 2019.
42. Government of Zimbabwe: **Harare Provincial Profile**. In. Harare: Parliament; 2011.
43. Ferrand R: **Unpublished data from the the Zimbabwe Study for Enhancing Testing and Improving Treatment of HIV in Children (ZENITH) - Individual questionnaire: Prevalence Survey https://doi.org/10.1371/journal.pmed.1002360.s006**. In.; 2016.
44. Rukuni R, McHugh G, Majonga E, Kranzer K, Mujuru H, Munyati S, Nathoo K, Gregson CL, Kuper H, Ferrand RA: **Disability, social functioning and school inclusion among older children and adolescents living with HIV in Zimbabwe**. *Tropical Medicine and International Health* 2017.
45. Simms V, Dauya E, Dakshina S, Bandason T, McHugh G, Munyati S, Chonzi P, Kranzer K, Ncube G, Masimirembwa C *et al*: **Community burden of undiagnosed HIV infection among adolescents in Zimbabwe following primary healthcare-based provider-initiated HIV testing and counselling: A cross-sectional survey**. *PLOS Medicine* 2017, **14**(7):e1002360.
46. Craig CL, Marshall AL, Sjöström M, Bauman AE, Booth ML, Ainsworth BE, Pratt M, Ekelund U, Yngve A, Sallis JF *et al*: **International physical activity questionnaire: 12-country reliability and validity**. *Med Sci Sports Exerc* 2003, **35**(8):1381-1395.
47. Filteau S, Rehman AM, Yousafzai A, Chugh R, Kaur M, Sachdev HPS, Trilok-Kumar G: **Associations of vitamin D status, bone health and anthropometry, with gross motor development and performance of school-aged Indian children who were born at term with low birth weight**. *BMJ Open* 2016, **6**(1).
48. FANTA: **Developing and Validating Simple Indicators of Dietary Quality of Infants and Young Children in Developing Countries: Additional analysis of 10 data sets. Report submitted to the Food and Nutrition Technical Assistance Project.** In. Edited by Indicators. WGoIaYCF. Washington, D.C.; 2007.
49. Foster HE, Jandial S: **pGALS - paediatric Gait Arms Legs and Spine: a simple examination of the musculoskeletal system**. *Pediatr Rheumatol Online J* 2013, **11**(1):44.
50. Crespi CM, Alfonso VH, Whaley SE, Wang MC: **Validity of child anthropometric measurements in the Special Supplemental Nutrition Program for Women, Infants, and Children**. *Pediatric research* 2012, **71**(3):286-292.
51. Marshall WA, Tanner JM: **Variations in pattern of pubertal changes in girls**. *Arch Dis Child* 1969, **44**(235):291-303.
52. Marshall WA, Tanner JM: **Variations in the pattern of pubertal changes in boys**. *Arch Dis Child* 1970, **45**(239):13-23.

For peer review only - http://bmjopen.bmj.com/site/about/guidelines.xhtml

53. Biver E, Calmy A, Delhumeau C, Durosier C, Zawadynski S, Rizzoli R: **Microstructural alterations of trabecular and cortical bone in long-term HIV-infected elderly men on successful antiretroviral therapy**. *AIDS* 2014, **28**(16):2417-2427.
54. SAMSON: **the Sub-Saharan African MuSculOskeletal Network (SAMSON)**. *https://thesamsonorg* 2019.
55. Compston J E: **Osteoporosis Review**. *Clinical Endocrinology* 1990, **33**(5):653-682.
56. Clark EM, Ness AR, Tobias JH: **Bone fragility contributes to the risk of fracture in children, even after moderate and severe trauma**. *J Bone Miner Res* 2008, **23**(2):173-179.
57. Washington Group on Disability Statistics, UNICEF: **Module on Child Functioning and Disability** *Available online from http://wwwwashingtongroup-disabilitycom/wp-content/uploads/2016/02/wg_unicef_child-disability-background-documentpdf* 2014.
58. **The WHO child growth standards.Growth reference, 5–19y.** [Geneva, Switzerland: World HealthOrganization; 2007 http://www.who.int/childgrowthref/en/13]
59. Häger-Ross C, Rösblad B: **Norms for grip strength in children aged 4-16 years**. *Acta Paediatr* 2002, **91**(6):617-625.
60. Armstrong M: **Youth Fitness Testing in South African Primary School Children: National Normative Data, Fitness and Fatness, and Effects of Socioeconomic Status**. Cape Town: University of Cape Town; 2009.

For peer review only

For peer review only - http://bmjopen.bmj.com/site/about/guidelines.xhtml

FIGURES AND TABLES

Figure 1. Hypothesized changes in bone mass across the life-course in HIV-infected and uninfected individuals - modified from Compston 1990 [55] and Arpadi 2014 [13]

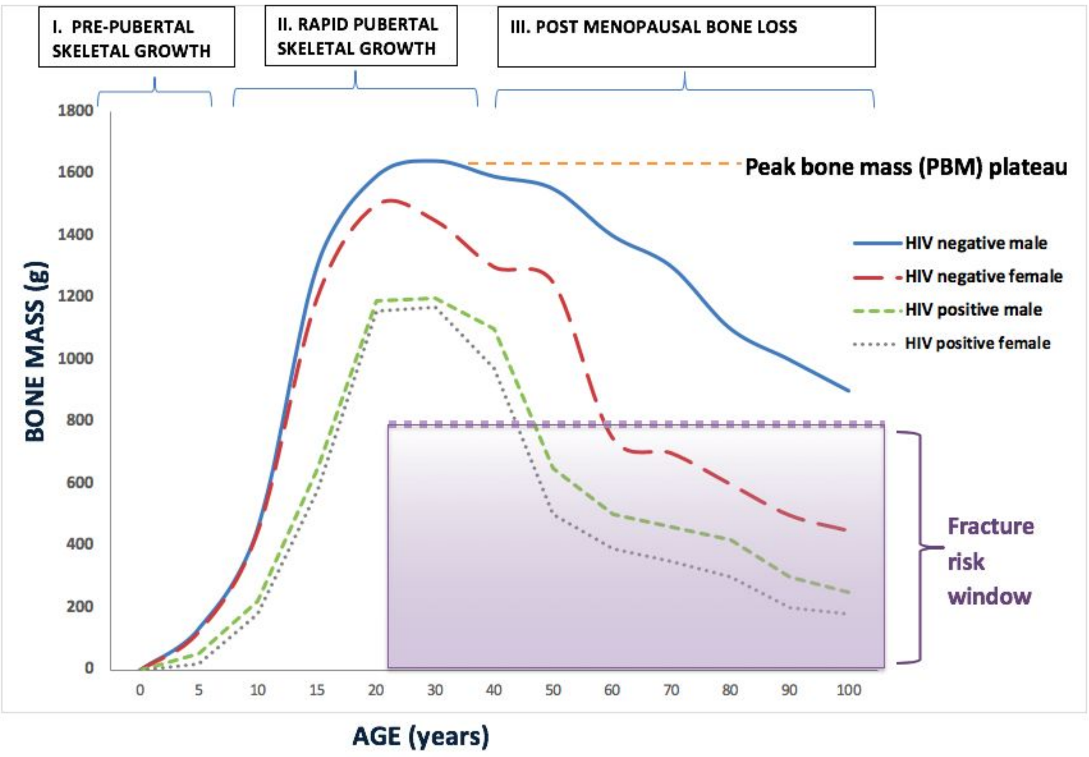

For peer review only - http://bmjopen.bmj.com/site/about/guidelines.xhtml

Figure 2. Hypothesised growth scenarios to be assessed as interactions between pubertal stage and HIV status on change in bone mass

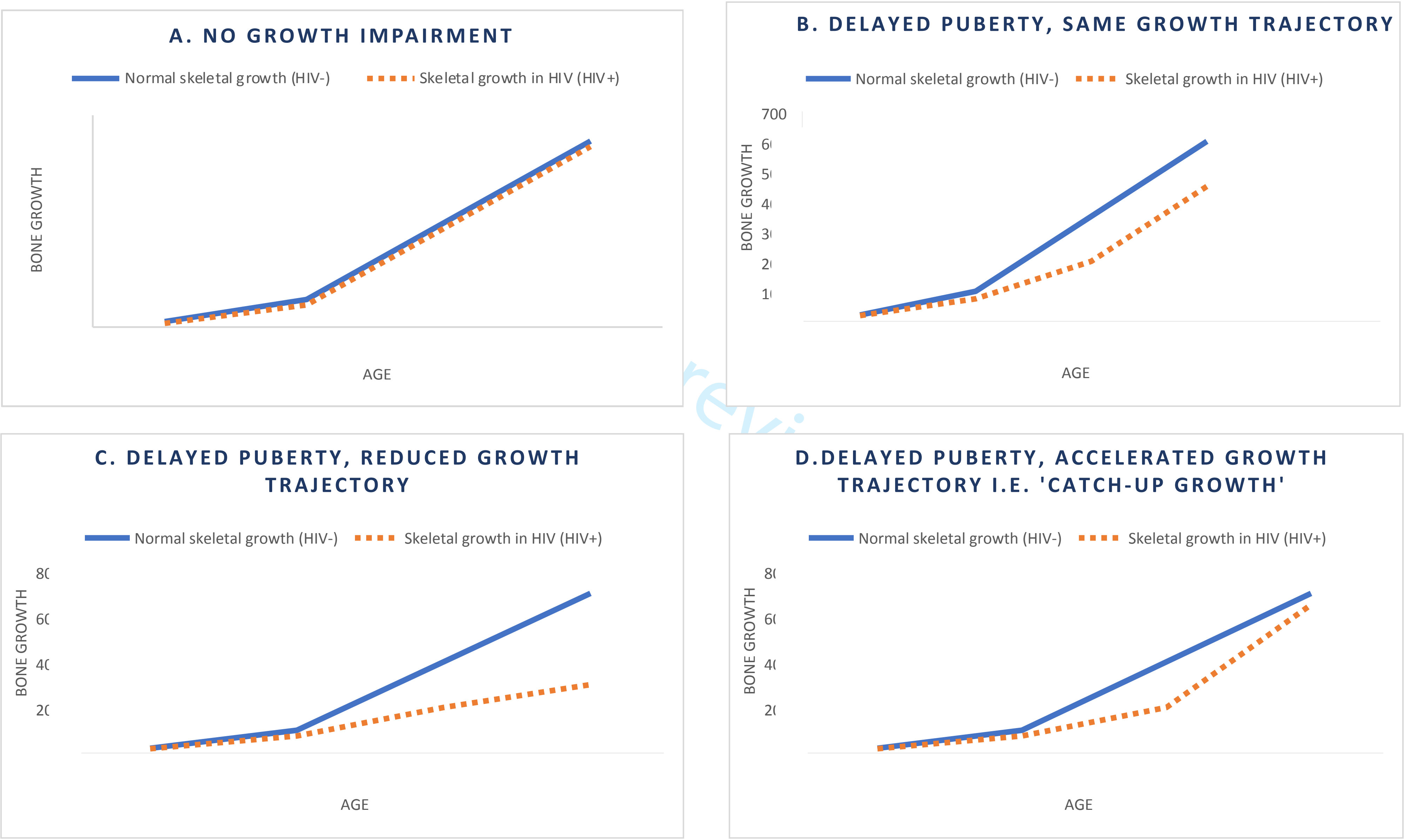

For peer review only - http://bmjopen.bmj.com/site/about/guidelines.xhtml

Table 1. Summary of study measurements to be quantified at baseline and follow-up

	Measurement	Measurement method	Outcome
INTERVIEW BASED QUESTIONNAIRE	Socio-demographic characteristics	Questionnaire	Age, sex, school attendance, orphanhood, guardianship
	Clinical history	Questionnaire[a]	History of fractures and trauma (modified Landin classification [56])<br>*HIV history: age at diagnosis, WHO disease stage, nadir CD4 count, opportunistic infections<br>*ART regimen/duration,<br>Exposures: steroid use, smoking, alcohol, recreational drugs<br>Family history of musculoskeletal disease & fractures<br>Other co-morbidities
	Physical activity	The International Physical Activity Questionnaire (IPAQ) [46] questionnaire (short form)	Median MET-minutes[b] of physical activity/week<br>1. inactive (<600 MET-minutes/week)<br>2. minimally active (600-1499 MET-minutes/week)<br>3. highly active (≥1500 MET-minutes/week)
	Nutrition[b]	Dietary assessment tool (Modified short food frequency questionnaire [47])	Daily dietary calcium and vitamin D intake<br>Prevalence of vitamin supplementation<br>Sun exposure
	Quality of life and disability	Washington Disability Score [57]	Functioning and disability score
STANDARDISED EXAMINATION	Musculoskeletal examination	Paediatric Gait Arms Legs and Spine (pGALS)[49] +/- regional clinical examination	Joint, spine and gait abnormalities
	Pubertal stage	Tanner's staging [51, 52]	Pre-pubertal (Stage 1)<br>Pubertal (Stage 2-3)<br>Post-pubertal (Stage 4 & 5)
	Anthropometry	Height (standing & sitting)<br>Weight<br>Mid-upper arm circumference (MUAC)[c]	Standing height-for-age (Z-score) [58][d]<br>Weight-for-age (Z-score) [58][d]<br>Body Mass Index (BMI) (Z-score) [58][d]<br>MUAC (Z-score) [58][d]
	Muscle strength	Jamar Dynamometer<br>Standing long jump[d]	Hand grip strength (kg, Z-score) [59][d]<br>Jumping distance (cm, Z-score) [60][e]
RADIOLOGY	Skeletal maturity	Hand/ wrist radiograph	Bone age (years)
	Bone and muscle composition	Dual-energy X-ray absorptiometry (DXA) of total body, lumbar spine and hip	Size corrected DXA measures of TBLH BMC^{LBM} (g), LS BMAD (g/cm^3) and Z-scores <-2.[d]<br>Lean mass
	Bone architecture	Peripheral quantitative computed tomography (pQCT)	Trabecular and cortical vBMD (g/cm^3),<br>Total and cortical CSA (mm^2), cortical thickness (mm), Periosteal and endosteal circumference (mm), SSI (units) PMI(mm^3) and CSMI (units)
BLOOD TESTS	Bone markers and DNA	Blood test (DNA extraction and serum saved)	Future testing
	HIV markers	Blood test	*CD4 count, HIV viral load

For peer review only - http://bmjopen.bmj.com/site/about/guidelines.xhtml

Table 1. Footnotes

a) Details of treatment and co-morbidities will be confirmed by patient-held medical records where available. **b)** Energy requirements defined in METS (multiples of the resting metabolic rate that give a score in MET-minutes). **c)** Nutritional indicator to include composite information from history (usual diet last month, sun exposure- vitamin D status) and clinical exam (MUAC). Similar methods have been used in other low income contexts [47]. **d)** Age and sex specific Z-scores for 1) *anthropometric measures:* will be determined using WHO child growth standards [58]; 2) hand *grip strength*: will be determined with reference to the uninfected comparison group and European normative data [59]; 3) *jumping distance:* will be determined using normative data from South Africa [60] 4) *low BMD* will be determined with reference to published paediatric Hologic DXA reference databases for LS BMAD and TBLH BMC^{LBM} Z-scores [36]. **e)** Standing long jump; the longest distance after two attempts will be recorded. **f)** Pregnancy urine dipstick in females prior to DXA if uncertain pregnancy status. **g)** Tests to be carried out on stored blood when further funding is secured.

*Denotes assessments to be carried out in HIV-infected participants only. **Abbreviations:** CSA (cross-sectional area), CSMI (cross sectional moment of inertia), LS BMAD (lumbar spine bone mineral apparent density) PMI (polar moment of inertia), SSI (Strength Strain Index), TBLH BMC^{LBM} (total-body less-head bone mineral content for lean mass adjusted for height).

For peer review only

For peer review only - http://bmjopen.bmj.com/site/about/guidelines.xhtml

The IMpact of Vertical HIV infection on child and Adolescent Skeletal development in Harare, Zimbabwe (IMVASK Study): a protocol for a prospective cohort study

Journal:	*BMJ Open*
Manuscript ID	bmjopen-2019-031792.R1
Article Type:	Protocol
Date Submitted by the Author:	21-Oct-2019
Complete List of Authors:	Rukuni, Ruramayi; London School of Hygiene and Tropical Medicine, Clinical Research Department; Biomedical Research and Training Institute, Harare<br>Gregson, Celia; University of Bristol, Musculoskeletal Research Unit; Royal United Hospital NHS Trust, Older Person's Unit<br>Kahari, Cynthia; London School of Hygiene and Tropical Medicine, 4. Department of Infectious Disease Epidemiology, Faculty of Epidemiology and Population Health; Biomedical Research and Training Institute (BRTI)<br>Kowo, Farirayi; University of Zimbabwe, Department of Radiology<br>McHugh, Grace; Biomedical Research and Training Institute, Harare<br>Munyati, Shungu; Biomedical Research and Training Institute, Harare<br>Mujuru, Hilda; University of Zimbabwe , College of Health Sciences<br>Ward, Kate; MRC Lifecourse Epidemiology Unit<br>Filteau, Suzanne; London School of Hygiene & Tropical Medicine, Population Health<br>Rehman, Andrea; London School of Hygiene and Tropical Medicine, Infectious Disease Epidemiology<br>Ferrand, Rashida; London School of Hygiene and Tropical Medicine
**Primary Subject Heading**:	Public health
Secondary Subject Heading:	Paediatrics, Radiology and imaging
Keywords:	HIV & AIDS < INFECTIOUS DISEASES, Tropical medicine < INFECTIOUS DISEASES, Paediatric radiology < PAEDIATRICS, Epidemiology < TROPICAL MEDICINE

I, the Submitting Author has the right to grant and does grant on behalf of all authors of the Work (as defined in the below author licence), an exclusive licence and/or a non-exclusive licence for contributions from authors who are: i) UK Crown employees; ii) where BMJ has agreed a CC-BY licence shall apply, and/or iii) in accordance with the terms applicable for US Federal Government officers or employees acting as part of their official duties; on a worldwide, perpetual, irrevocable, royalty-free basis to BMJ Publishing Group Ltd ("BMJ") its licensees and where the relevant Journal is co-owned by BMJ to the co-owners of the Journal, to publish the Work in this journal and any other BMJ products and to exploit all rights, as set out in our licence.

The Submitting Author accepts and understands that any supply made under these terms is made by BMJ to the Submitting Author unless you are acting as an employee on behalf of your employer or a postgraduate student of an affiliated institution which is paying any applicable article publishing charge ("APC") for Open Access articles. Where the Submitting Author wishes to make the Work available on an Open Access basis (and intends to pay the relevant APC), the terms of reuse of such Open Access shall be governed by a Creative Commons licence – details of these licences and which Creative Commons licence will apply to this Work are set out in our licence referred to above.

Other than as permitted in any relevant BMJ Author's Self Archiving Policies, I confirm this Work has not been accepted for publication elsewhere, is not being considered for publication elsewhere and does not duplicate material already published. I confirm all authors consent to publication of this Work and authorise the granting of this licence.

For peer review only

The IMpact of Vertical HIV infection on child and Adolescent Skeletal development in Harare, Zimbabwe (IMVASK Study): a protocol for a prospective cohort study

Ruramayi Rukuni[1,2], Celia L Gregson[3], Cynthia Kahari[2,4], Farirayi Kowo[5], Grace McHugh[2], Shungu Munyati[2], Hilda Mujuru[6], Kate A Ward[7], Suzanne Filteau[8], Andrea M Rehman[4] and Rashida A Ferrand[1,2]

Affiliations:

1. Clinical Research Department, Faculty of Infectious and Tropical Diseases, London School of Hygiene and Tropical Medicine (LSHTM), London, UK.
2. Biomedical Research and Training Institute (BRTI), Harare, Zimbabwe.
3. The Musculoskeletal Research Unit, Translational Health Sciences, Bristol Medical School, University of Bristol, Bristol, UK.
4. Department of Infectious Disease Epidemiology, Faculty of Epidemiology and Population Health, London School of Hygiene and Tropical Medicine (LSHTM), London, UK.
5. Department of Radiology, University of Zimbabwe, Harare, Zimbabwe.
6. Department of Paediatrics, University of Zimbabwe, Harare, Zimbabwe.
7. MRC Lifecourse Epidemiology Unit, University of Southampton, Southampton, UK.
8. Department of Population Health, Faculty of Epidemiology and Population Health, London School of Hygiene and Tropical Medicine (LSHTM), London, UK.

Corresponding Author

Dr Ruramayi Rukuni

Biomedical Research and Training Institute (BRTI), 10 Seagrave Rd, Avondale, Harare, Zimbabwe.

Tel: +263 719 362 961

Email: Ruramayi.Rukuni@lshtm.ac.uk

For peer review only

For peer review only - http://bmjopen.bmj.com/site/about/guidelines.xhtml

ABSTRACT

Introduction

The scale-up of antiretroviral therapy (ART) across sub-Saharan Africa (SSA) has reduced mortality so that increasing numbers of children with HIV (CWH) are surviving to adolescence. However, they experience a range of morbidities due to chronic HIV infection and its treatment. Impaired linear growth (stunting), is a common manifestation, affecting up to 50% of children. However, the effect of HIV on bone and muscle development during adolescent growth is not well characterised. Given the close link between pubertal timing and musculoskeletal development, any impairments in adolescence are likely to impact on future adult musculoskeletal health. We hypothesize that bone and muscle mass accrual in CWH is reduced, putting them at risk of reduced bone mineral density (BMD) and muscle function and increasing fracture risk. This study aims to determine the impact of HIV on BMD and muscle function in peri-pubertal children on ART in Zimbabwe.

Methods and analysis

CWH (n=300) and without HIV (n=300), aged 8-16 years, established on ART, will be recruited into a frequency-matched prospective cohort study and compared. Musculoskeletal assessments including dual-energy X-ray absorptiometry (DXA), peripheral quantitative computed tomography (pQCT), grip strength and standing long jump will be conducted at baseline and after one year. Linear regression will be used to estimate mean size-adjusted bone density and Z-scores by HIV status (*i.e.* total-body less-head (TBLH) bone mineral content (BMC) for lean mass adjusted for height (TBLH BMC^{LBM}) and lumbar spine bone mineral apparent density (LS BMAD). The prevalence of low size-adjusted BMD (i.e. Z-scores <-2) will also be determined.

Ethics and dissemination

Ethical approval for this study has been granted by the Medical Research Council of Zimbabwe and the LSHTM Ethics Committee. Baseline and longitudinal analyses will be published in peer reviewed journals and disseminated to research communities.

For peer review only - http://bmjopen.bmj.com/site/about/guidelines.xhtml

STRENGTHS AND LIMITATIONS OF THIS STUDY

- This study will provide novel understanding of the effects of HIV on bone and muscle development in a large population of sub-Saharan African (SSA) children living with HIV by using 'gold standard' size adjustment methods for DXA, which are crucial for assessing a population with inherent size differences
- This prospective study will provide understanding of how bone and muscle change over time
- Bone architecture measurement by pQCT will provide understanding of trabecular and cortical bone geometry and strength in CWH
- This study will generate new data for total body and lumbar spine DXA, tibial pQCT, hand grip strength and standing long jump for Zimbabwean children without HIV which will inform normative reference data
- Whilst the age range in this study, 8-16 years, will allow analysis of pubertal delay in children with HIV, the follow-up period is insufficient to determine the impact on attainment of peak bone mass

INTRODUCTION

Sub-Saharan Africa (SSA) disproportionally bears the burden of global HIV infection, with nearly 90% of the estimated 2.1 million children under 15 years of age living in SSA [1]. The global scale-up of antiretroviral therapy (ART) has dramatically improved survival of children with HIV (CWH) [2]. However there is accumulating evidence that the growing number of these children are now reaching adolescence in SSA with multisystem chronic comorbidities associated with HIV infection and/or its treatment [3].

Poor linear growth (*i.e.* stunting), is one of the most common manifestations of perinatally-acquired HIV infection, affecting up to 50% of children [4, 5]. Linear growth is greatest in adolescence during the pubertal development period. Bone mass is thought to change throughout the life course and may be altered by HIV [6, 7] (Figure 1). The majority of peak bone mass (PBM), the maximum amount of bone accrued by the end of skeletal maturation, is attained during adolescent growth; by age 18 years in women and age 20 years in men, 80% of PBM is attained [8]. After PBM is reached, there is no net gain in bone mass. Therefore PBM is the net reservoir of bone for later life, a key determinant of adult bone mineral density (BMD) and consequently of adult osteoporotic fracture risk [9]. Linear growth is therefore intimately linked to skeletal development but how HIV infection affects bone development in peri-pubertal SSA children is largely unknown. The prevalence of low BMD has been found to be higher in CWH than uninfected children in high and middle income countries (7% in the USA [10], 32%

For peer review only - http://bmjopen.bmj.com/site/about/guidelines.xhtml

in Brazil [11] and 24% in Thailand [12] compared to 1% in children without HIV in the USA [10]. No study has estimated the prevalence of low BMD in SSA, and the prevalence of and risk factors for low BMD in African CWH is not known [6, 13]. It is important to highlight that the risk of poor bone accrual, reflected in low BMD measurements, is likely to be different in low income countries compared to high income countries due to factors such as malnutrition and social deprivation; but critically due to delayed ART initiation. A recent meta-analysis has shown that the median age of ART initiation in the UK/USA is two years, compared to eight years in SSA [14].

The mechanisms by which HIV may lead to low size-adjusted BMD in children are not fully understood but are likely multifactorial including HIV-associated factors (*e.g.* ART drugs, HIV disease stage) and traditional risk factors (*e.g.* hypogonadism, smoking, alcohol, low physical activity and vitamin D deficiency) [15]. HIV infection promotes systemic immune activation and production of inflammatory cytokines (*e.g.* TNFα) that in turn promote increased bone resorption [16]. ART initiation, particularly with tenofovir (part of the first-line ART regimen in SSA), predicts an initial decline in BMD which stabilizes after two years in adults [17]. It is thought tenofovir may cause renal proximal tubule toxicity resulting in phosphate wasting and increased bone turnover [18]. Although tenofovir and protease inhibitors have been associated with low BMD in adults [19, 20], studies in children have shown inconsistent findings [21-23]. Malnutrition, opportunistic infections and social deprivation may also impede musculoskeletal development. Reduced physical activity, associated with HIV [24], may also impair muscle development and limit impact loading to reduce osteocyte-mediated bone accrual [25, 26] . In adults, weak grip strength has been associated with increased falls and fracture risk [27]. Although muscle (lean) mass has also been shown to predict the magnitude of bone accrual during growth [28], few studies have compared muscle strength and function between children with and without HIV. Interestingly, a small Canadian study showed deficits in muscle power in CWH [29].

Another mechanism by which HIV may exert effects on BMD is through its effect on puberty. Even in the presence of ART, the onset of puberty is delayed by approximately a year in CWH in both high income [30] and low income settings [31]. Older age at ART initiation has been shown to be a significant risk factor for pubertal delay in Zimbabwean CWH [31]. Pubertal delay in HIV may be mediated through nutritional deficiency, recurrent infection, or chronic immune activation disrupting hormonal regulation [31]. Delayed puberty may be advantageous for linear growth; spending more time in puberty may allow more time for skeletal growth [31]. Conversely, delayed puberty has been shown in studies in high income settings to be detrimental to bone mass accrual [32, 33]. However, the impact of pubertal delay on BMD in low income countries remains unknown. Pubertal delay can be assessed objectively using hand radiographs. Analysis of the growth plate development and fusion

For peer review only - http://bmjopen.bmj.com/site/about/guidelines.xhtml

of long bones in the hands can accurately quantify bone age, which is a measure of skeletal maturation. Bone age lagging behind chronological age reflects pubertal delay [34].

BMD is commonly measured by Dual-energy X-ray absorptiometry (DXA) as two-dimensional (areal) BMD, however, this is highly dependent on bone size [35]. DXA underestimates areal bone density in short children, with smaller bones, and overestimates BMD in taller children, with bigger bones, despite the fact that they may have identical volumetric BMD. Size adjustment of DXA measures is therefore critically important in children with chronic diseases such as HIV, where smaller size due to poorer growth and delayed puberty may explain findings of lower BMD. The two 'gold standard' size-adjustment techniques chosen from the International Society for Clinical Densitometry (ISCD) are: bone mineral apparent density at the lumbar spine (LS BMAD) and regression based total-body less-head (TBLH) Bone Mineral Content (BMC) for lean mass adjusted for height (TBLH BMC^{LBM}) [36] Z-scores. As there are currently no published reference DXA data for child or adolescent populations in SSA, in this study we will use the of best available data sets from high income countries such as the UK [36] to generate Z-scores.

Unlike DXA, peripheral quantitative computed tomography (pQCT) takes into account bone size by directly measuring volumetric BMD. It has the additional advantage of separately assessing trabecular and cortical bone compartments, providing information on bone architecture. Furthermore, a range of bone strength indices *e.g.* strength stain index, validated against fracture risk can be calculated [37, 38]. In high income countries, markedly abnormal trabecular and cortical architecture have been shown in adults with HIV [39] and abnormal bone architecture and impaired bone strength through to early adulthood have been shown in boys with HIV infection [39]. Few studies have assessed bone architecture and strength in CWH in SSA.

The IMVASK study aims to determine the prevalence of low size-adjusted BMD and muscle function (grip strength and standing long jump) in Zimbabwean children with and without HIV. pQCT assessment will enable understanding of the impact of HIV infection on bone architecture and strength. This study will further contribute to local reference data for DXA measures, bone age and muscle function (grip strength and standing long jump) for a sub-Saharan African population, establishing a biorepository for future research. Study results will aid understanding of bone and muscle accrual in the context of HIV infection in the era of ART.

For peer review only - http://bmjopen.bmj.com/site/about/guidelines.xhtml

METHODS AND ANALYSIS

Study objectives

To determine the impact of HIV infection on size-adjusted bone density in peri-pubertal children aged 8-16 years established on ART. The objectives of this prospective study are:

1) To quantify the prevalence of low size-adjusted BMD and low muscle function (grip strength and standing long jump) among CWH compared to uninfected children
2) To investigate the risk factors for low size-adjusted bone density and low muscle function (grip strength and standing long jump) among children with HIV
3) To compare the rates of bone mass accrual over one year between children with and without HIV and assess for interaction by pubertal stage to determine if CWH exhibit catch up growth
4) To determine the differences in bone architecture measured by pQCT between children with and without HIV

Study hypothesis

We hypothesize that HIV infection adversely affects skeletal development, such that CWH, despite ART, accrue less bone mass and strength and have reduced muscle function during skeletal development.

Study design

CWH aged 8-16 years and established on ART (n=300) and a comparison group of children without HIV, frequency-matched for age and sex (n=300) will be recruited into a prospective cohort study. Detailed musculoskeletal assessments will be conducted at baseline and after one year.

Study setting

Parirenyatwa and Harare Hospital are the largest public-sector referral hospitals in Harare [40, 41]. The paediatric HIV clinics at both hospitals provide HIV care to more than 2,000 children. Although HIV care is increasingly decentralised to primary care level across the country, most children in Harare continue to receive care within HIV clinics in secondary healthcare facilities. Parirenyatwa hospital has a well-functioning radiology department which houses the University of Zimbabwe DXA and pQCT research unit and has access to private radiology services in the surrounding area. The hospital catchment areas have over 116 primary and 42 secondary government schools with an estimated 157,962 children enrolled [42]. School attendance in Harare province is high and does not differ by HIV status, with 96% of children under 18 years attending school [43].

For peer review only - http://bmjopen.bmj.com/site/about/guidelines.xhtml

Recruitment of participants

Eligibility

Inclusion criteria: age 8-16 years (includes pre- and peri-pubertal children), living in Harare, and in CWH only if:

i. perinatally-acquired HIV and taking ART for at least two years (as adult studies demonstrate ART initiation is followed by an initial decline in BMD which stabilizes after 2 years [17]).
ii. the child is aware of their HIV status, to avoid inadvertent disclosure as a result of study participation.

Exclusion criteria: acute illness (requiring immediate hospitalisation) and lack of consent.

Recruitment of children with HIV

Systematic quota-based sampling by age and sex will be used to recruit 300 children from Parirenyatwa and Harare Hospital HIV clinics. Participants will be recruited sequentially as they attend clinic such that 50 males and 50 females will be chosen for each of three age-strata, 8-10.99, 11-13.99 and 14-16.99 years. A maximum of 5 participants will be enrolled on each day for logistical reasons. The total number of children approached each day will be recorded, irrespective of whether they are subsequently eligible or enrolled to determine the sampling fraction. Written consent will be obtained from children and their guardians. Study processes and procedures will be clearly explained to children and their guardians and they will be given the option to accept or decline to take part in the research. It will also be explained that they are allowed to withdraw from the study at any time, for any reason, without affecting the care they receive from the clinic.

Recruitment of children without HIV

Three hundred CWH will be randomly sampled from six government primary and secondary schools in the same catchment area as Parirenyatwa and Harare Hospitals. Younger children (8-12 years) will be selected from primary schools and older children (13-16 years) from secondary schools, with thirteen-year olds coming from both primary and secondary schools. The number of children selected from each school will be proportional to school size, thereby giving each child equal probability of being sampled. A random number sequence will be generated, and school registers will be used to select participants of similar age and sex as the children with HIV using the same quota-based approach of 50 males and 50 females in each of the three age strata. Guardians of selected school children will be invited to the study clinic to complete the consent process. Consenting participants will have a diagnostic HIV test as part of their assessment. Those testing HIV positive (anticipated to be approximately 2-3% [44]) will be referred for HIV care.

For peer review only - http://bmjopen.bmj.com/site/about/guidelines.xhtml

For peer review only

Study procedures

Questionnaire

An interviewer-administered questionnaire together with hand-held medical records will be used to collect socio-demographic details and clinical history including age, sex, school attendance, orphan status, guardianship, history of fractures with mechanism of trauma, steroid use, smoking, alcohol, recreational drugs, family history of musculoskeletal disease, co-morbidities, physical activity, diet and nutrition and sun exposure. Where possible, validated instruments adapted for the local context will be used. For example, the International Physical Activity Questionnaire (IPAQ) [45] validated in multiple countries including South Africa and will be used to assess physical activity as multiples of the resting metabolic rate (MET) in MET-minutes. Diet and nutrition will be assessed using a tool we developed for the Zimbabwean context based on a validated dietary diversity and food frequency tool from India and Malawi [46] and international guidelines applicable to SSA [47]. The tool quantifies vitamin D supplementation and sunlight exposure and has been adapted to reflect the Zimbabwean context where fortification of oils and margarine with vitamin D is mandated by the government and specific vitamin D rich foods such as kapenta fish are found.

Clinical examination

A standardised musculoskeletal examination will be conducted using the validated paediatric gait, arms, legs and spine (pGALS) examination [48]. Additional clinical assessments will be carried out using standardised protocols and calibrated equipment. Anthropometry measurements will include standing and sitting height, arm span, mid upper arm circumference. Height will be measured to the nearest 0.1 cm, by two separate readers using calibrated Seca 213 stadiometers. If the two height measurements differ by more than 0.5 cm , a third reading will be taken [49]. Weight will be measured to the nearest 0.1 kg using calibrated Seca 875 scales. Tanner pubertal staging will be carried out using a standardised protocol with an orchidometer to assess testicular volume in males [50]. Muscle function will be assessed in the upper limb and lower limbs by grip strength dynamometry and standing long jump respectively. Hand grip strength will be measured using a Jamar hydraulic hand-held dynamometer (Patterson Medical, UK) to the nearest 0.1kg. Participants will be seated with the shoulder at 0° to 10°, the elbow at 90° of flexion and the forearm positioned neutrally. Three measurements will be taken from each hand in alternation and the highest measurement chosen. The standing long jump distance will be taken from the best of three correctly performed attempts to the nearest 0.1 cm, measuring the distance from the take-off line to the heel.

For peer review only - http://bmjopen.bmj.com/site/about/guidelines.xhtml

Radiological assessments

DXA scans will be performed by two trained radiographers using a Hologic QDR Wi densitometer with Apex software version 4.5. Measurements will be taken from the lumbar spine, hip and total body. Fat and muscle mass will also be acquired; muscle mass is the fat free mass measurement from DXA. DXA scans will be repeated in a subgroup (n=20) of participants to determine reproducibility. pQCT measurements of the non-dominant tibia will be taken using a Stratec XCT-2000 scanner (Stratec, Pforzheim, Germany) software version 6.20. Measurements of the non-dominant tibia will be taken at three sites at 4%, 38%, and 66% percent of the tibial length, measured from the medial malleolus to the medial tibial plateau. Daily quality control will be performed by scanning the manufacturer provided lumbar spine phantom for DXA and tibia phantom for pQCT. A radiograph of the non-dominant hand and wrist will be taken and used to quantify bone age using the Greulich and Pyle (G&P) atlas and the Tanner Whitehouse 3 (TW3) method. For Intra-observer reliability, 10% of the radiographs will be randomly selected and rescored by the same operator after one week. For inter-observer reliability a different set of 10% of the radiographs will be re-scored by a different expert. The estimated bone age will then be compared to the calculated chronological age.

Blood tests

A fasting blood sample (up to 15ml) will be collected. HIV markers (CD4 count and viral load) will be tested in CWH only. CD4 cell count will be measured using an Alere PIMA CD4 machine (Waltham, Massachusetts, USA). HIV viral load will be measured using the GeneXpert HIV-1 viral load platform (Cepheid Inc, Sunnyvale, California, USA). The remaining blood plasma will be bio-banked to enable future measurement of bone biochemistry. After removing the plasma, peripheral blood mononuclear cells (PBMC) will be isolated and cryopreserved. DNA will also be extracted using a manual method and stored for future genetic studies.

Follow up at one year

All study measurements, with the exception of DNA extraction, will be repeated after one year. Participants will be recalled exactly one year after their first DXA scan. The aim is to perform all scans within a 4 week window period. Contact will be maintained with participants via regular phone calls and text messaging to minimise loss-to-follow-up. The schedule of study procedures is summarised in Table 1.

Outcome measures

The primary study outcomes are:

1) mean size-adjusted bone density Z-scores; TBLH BMC^{LBM} and LS BMAD [36].
2) the prevalence of low TBLH BMC^{LBM} and LS BMAD Z-score <-2 at baseline [36].

For peer review only - http://bmjopen.bmj.com/site/about/guidelines.xhtml

Secondary study outcomes are:

1) prevalence of low muscle function; grip strength and standing long jump-for-age (Z-score<-2) and musculoskeletal abnormalities/disabilities by HIV status at baseline.
2) mean percentage change in TBLH BMC^{LBM} (g) and LS BMAD (g/cm^3), tibial cortical and trabecular volumetric BMD (g/cm^3), total cross sectional area, cortical thickness and bone strength, muscle mass and function at baseline and one year, by HIV status.
3) assessment of the extent to which pubertal delay explains changes in these bone and muscle outcomes.

Sample size

The sample size was calculated to detect differences in DXA-measured mean size-adjusted bone BMD Z-scores between children with and without HIV. This study will have 80% power (α 0.05) to detect a 0.23 Z-score difference between 300 HIV-infected and 300 uninfected children, assuming a standard deviation of 1.3. As there were no published studies from low income countries, estimates of the expected difference were taken from a US study of children with HIV aged 7 to 15 years [10]. In addition, our study will have 80% power to detect a 4.8% difference in the prevalence low size-adjusted BMD between the two groups. This is a smaller prevalence difference than that detected by the most conservative prevalence estimate of low BMD of 7% from three studies in high and middle-income countries [10-12].

Statistical analysis

For continuous variables with normally distributed residuals, the mean and standard deviation will be presented. For skewed continuous variables, the median and inter-quartile range (IQR) will be presented. Categorical variables will be summarised as frequencies and percentages. The distribution of demographic and clinical variables will be compared between CWH and without HIV using *t*-tests for means, Wilcoxon rank sum test for medians and Chi-squared tests for proportions.

Baseline mean TBLH BMC^{LBM} and LS BMAD Z-scores and the prevalence of low TBLH BMC^{LBM} and LS BMAD Z-score will be compared between CWH and without HIV. Among CWH, the association between *a priori* defined risk factors (ART duration, ART type, proportion of life on treatment, age at ART initiation, CD4 count, viral load, bone age, pubertal stage, nutrition, socioeconomic status and orphanhood) against size-adjusted BMD will be examined using multivariable linear regression (Z-score as a continuous variable) and multivariable logistic regression (as defined by the Z-score cut off of <-2).

For peer review only - http://bmjopen.bmj.com/site/about/guidelines.xhtml

For peer review only

Paired sample t test or nonparametric Wilcoxon test will be used to assess for differences in TBLH BMC and LS BMAD on CWH and children without HIV between baseline and follow up. Multivariable linear regression will be used to analyse the mean percentage change in TBLH BMC^{LBM}(g) and LS BMAD (g/cm^3) between children with and without HIV. Models will be adjusted for physical activity, calcium and vitamin D intake. Interaction between the effects of pubertal stage (bone age) and HIV on change in TBLH BMC^{LBM} and LS BMAD will be investigated to see if differences in bone density become more or less pronounced through puberty *i.e*. whether catch-up growth is possible, see Figure 2. The regression coefficient (β) for percentage change in size-adjusted bone mass may suggest either no growth impairment (Figure 2A), delayed puberty whilst maintaining the same growth trajectory (Figure 2B) or delayed puberty with a reduced growth trajectory (Figure 2C) in CWH. If β is markedly more positive in CWH, this suggests that catch-up growth may be possible (Figure 2D). Pubertal delay in this study will be defined as the lack of the initial signs of puberty (Tanner stage 2) at an age that is more than 2 standard deviations beyond the population mean [51] and as chronological age minus bone age > 2 years [52]. Data for total body and lumbar spine DXA, tibial pQCT, hand grip strength and standing long jump in CWH will be analysed with reference to the comparator group of children without HIV.

For the purposes of normative data derivation, children without HIV who have any diagnosis or evidence of muscle or bone disease will be excluded. Then outliers with bone density, hand grip strength or standing long jump data beyond 2 standard deviations from the mean will have their case record reviewed to exclude cases with underlying bone or muscle pathology. The remaining population will be used to generate normative references ranges for these quantitative traits.

Data management

Data collection, management and storage will be governed by standard operating procedures and will follow the principles of Good Clinical Practice (GCP). Data will be captured using hand held tablets for the questionnaires. Paper forms will be available in case of failure of electronic data entry. Microsoft Access will be used as the main backend database as it allows programming of quality control checks and conditional data validation. GCP compliant audit trail modules will be incorporated into the databases and reports of aggregated data will be reviewed on a monthly basis. In order to assure data quality and consistency, all staff will receive regular training and regular quality checks will be conducted. Paper records will be stored for eight years after the completion of research in secure, locked storage facilities. Field staff will download data to the central database, which is backed up onto an encrypted external hard drive daily, and to additional off-site and secure cloud back-up. The off-site back-up copies will be stored through the London School of Hygiene and Tropical Medicine

For peer review only - http://bmjopen.bmj.com/site/about/guidelines.xhtml

(LSHTM) Research Data Management Support Service that has an established data repository. In order to preserve the long-term value of this data, it will be stored backed-up here indefinitely. Anonymised research data will be made available for sharing through the open access data repository established by the LSHTM Data Management Support Service at the time of publication. This will allow other research groups to request access to study data and tools. Information on how other researchers' data will be included in every study publication.

Patient and Public Involvement

Whilst patients were not directly involved in the design and conduct of the study, feedback from patient experiences in the study will be used to inform planned public engagement activities, which include science fairs, conducted by the research team at schools from where participants were recruited.

Study status

Recruitment to this study began in May 2018 and is planned until August 2019. Study follow up will run from May 2019 to August 2020.

DISCUSSION

Although the scale-up of prevention of mother-to-child transmission (PMTCT) has reduced perinatal HIV transmission but coverage is still not universal in most parts of SSA and therefore perinatal HIV infection is expected to affect large numbers of children for years to come. Furthermore, the scale-up of ART has reduced HIV-associated mortality dramatically so that CWH, who would previously have died in infancy or early childhood, are now reaching adolescence in increasing numbers. It is therefore important to understand the impact of HIV infection and its treatment on skeletal development during the critical period of puberty.

This study will determine the prevalence of low size-adjusted BMD in children with and without HIV in Zimbabwe, a country with a severe sustained early onset HIV epidemic. In addition, this study will determine risk factors for low size-adjusted BMD in CWH. We aim to identify factors amenable to intervention, which may be modifiable to maximize future bone health and minimize subsequent adult osteoporotic fracture risk. For example, reduced muscle function predicting low size-adjusted BMD, may suggest targeted physiotherapy would be of benefit which would warrant formal investigation.

Our study will provide insights regarding the mechanisms through which perinatal HIV infection affects the timing of pubertal onset and bone mass accrual. By measuring bone and muscle parameters at

For peer review only - http://bmjopen.bmj.com/site/about/guidelines.xhtml

baseline and one year and employing 'gold standard' size-adjustment methodology for DXA-measured BMD in the growing skeleton, this study will also provide insights into whether catch-up growth in terms of bone mass accrual is possible in HIV despite pubertal delay and provide age-related growth velocity data for CWH, with and without puberty. Whilst the age range in this study, will allow analysis of pubertal delay in CWH, the follow-up period is insufficient to determine the impact on attainment of peak bone mass. An additional limitation is that it will not be possible to obtain accurate height data for CWH prior to enrolment in order to fully study growth recovery.

The bone architecture measured by pQCT in this study will provide separate assessments of trabecular and cortical bone density, and bone geometry and strength in Zimbabwean children. The evidence from studies in adult men established on ART demonstrate impairments in trabecular and cortical bone architecture [53]. Whether the same applies to children needs to be determined.

Furthermore, we will establish novel comparator data for DXA, pQCT, bone age, hand grip strength and standing long jump for a Zimbabwean population, which will be able to be used for future research in this context. This study will establish a biorepository for future research *e.g.* potential bone turnover marker measurement and genotyping.

Given the magnitude of the HIV epidemic in SSA and the large cohort of young people who may experience impaired bone accrual, musculoskeletal disability or fracture as they reach adolescence and early adulthood; it is imperative to characterise the impact of perinatal HIV on musculoskeletal development.

ETHICS AND DISSEMINATION

Ethical approval has been granted by the London School of Hygiene and Tropical Medicine Ethics Committee (Ref: 15333; 14 May 2018), the Institutional Review Board of the Biomedical Research and Training Institute (Ref: AP 145/2018; 20 February 2018), the Joint Research Ethics Committee for University of Zimbabwe College of Health Sciences and the Parirenyatwa Group of Hospitals (JREC) (Ref: 11/18; 1 March 2018), Harare Central Hospital Ethics Committee (HCHEC) (Ref: 170118/04; 23 February 2018), the Medical Research Council of Zimbabwe Ref: (MRCZ/A/2297; 10 April 2018) and the Ministry of Primary and Secondary Education Zimbabwe (Ref: C/426/Harare; 13 February 2018). This study is registered with the ISRCTN registry (Ref: ISRCTN12266984)

Study progress will be reported annually to MRCZ. Results of interim data analysis will be presented at national and international research meetings and conferences. Study findings will be published in

For peer review only - http://bmjopen.bmj.com/site/about/guidelines.xhtml

international peer reviewed scientific journals and disseminated to research communities at the end of study.

AUTHORS' CONTRIBUTIONS

RR, RAF and CG co-designed the study. RR wrote the study protocol and was responsible for journal selection and preparation of the first draft of this article as the principal author. CK contributed to the development of the pQCT protocols. FK contributed to the development of the bone age analysis protocols. KW provided scan protocols, contributed to the study design, and gave methodological input regarding bone density size-adjustment and analysis. AR contributed to the study design, in particular, sampling strategy, sample size calculation and the statistical analysis plan. SF provided advice regarding the development of nutritional assessment tools. GM, SM and HM advised on study conduct and provided study oversight. All authors reviewed and provided feedback on the manuscript prior to submission.

FUNDING STATEMENT

This study is funded by the Wellcome Trust UK. RR is funded by Wellcome Trust UK grant number 206764/Z/17/Z. CK is funded by a NIH Fogarty Fellowship. RAF is funded by Wellcome Trust grant number 206316/Z/17/Z. Global challenges research funding from the University of Bristol established the Sub-Saharan African MuSculOskeletal Network (SAMSON) enabling the provision of pQCT in Zimbabwe for this study. AMR is additionally supported by the UK Medical Research Council (MRC) and the UK Department for International Development (DFID) under the MRC/DFID Concordat agreement which is also part of the EDCTP2 programme supported by the European Union grant reference (MR/R010161/1).

COMPETING INTERESTS STATEMENT

The authors have no competing interests to declare.

REFERENCES

1. UNICEF: **Monitoring the Situation of Children and Women; Global and regional trends, current status and progress.** https://datauniceforg/topic/hivaids/global-regional-trends/# 2017.
2. Celletti F, Sherman G, Mazanderani AH: **Early infant diagnosis of HIV: review of current and innovative practices**. *Curr Opin HIV AIDS* 2017, **12**(2):112-116.
3. Lowenthal ED, Bakeera-Kitaka S, Marukutira T, Chapman J, Goldrath K, Ferrand RA: **Perinatally acquired HIV infection in adolescents from sub-Saharan Africa: a review of emerging challenges**. *Lancet Infect Dis* 2014, **14**(7):627-639.

For peer review only - http://bmjopen.bmj.com/site/about/guidelines.xhtml

4. McGrath CJ, Chung MH, Richardson BA, Benki-Nugent S, Warui D, John-Stewart GC: **Younger age at HAART initiation is associated with more rapid growth reconstitution**. *AIDS* 2011, **25**(3):345-355.
5. WHO: **Growth failure in HIV-infected children**. In: *Consultation on Nutrition and HIV/AIDS in Africa: Evidence, lessons and recommendations for action.* Edited by SM A. Geneva, Switzerland: World Health Organisation, Department of Nutrition for Health and Development; 2005.
6. Arpadi SM, Shiau S, Marx-Arpadi C, Yin MT: **Bone health in HIV-infected children, adolescents and young adults: a systematic review**. *J AIDS Clin Res* 2014, **5**(11).
7. Compston J E: **Osteoporosis Review**. *Clinical Endocrinology* 1990, **33**(5):653-682.
8. Negredo E, Domingo P, Ferrer E, Estrada V, Curran A, Navarro A, Isernia V, Rosales J, Perez-Alvarez N, Puig J *et al*: **Peak bone mass in young HIV-infected patients compared with healthy controls**. *J Acquir Immune Defic Syndr* 2014, **65**(2):207-212.
9. Hernandez CJ, Beaupré GS, Carter DR: **A theoretical analysis of the relative influences of peak BMD, age-related bone loss and menopause on the development of osteoporosis**. *Osteoporos Int* 2003, **14**(10):843-847.
10. DiMeglio LA, Wang J, Siberry GK, Miller TL, Geffner ME, Hazra R, Borkowsky W, Chen JS, Dooley L, Patel K *et al*: **Bone mineral density in children and adolescents with perinatal HIV infection**. *AIDS* 2013, **27**(2):211-220.
11. Schtscherbyna A, Pinheiro MF, Mendonca LM, Gouveia C, Luiz RR, Machado ES, Farias ML: **Factors associated with low bone mineral density in a Brazilian cohort of vertically HIV-infected adolescents**. *International Journal of Infectious Diseases* 2012, **16**(12):e872-878.
12. Puthanakit T, Saksawad R, Bunupuradah T, Wittawatmongkol O, Chuanjaroen T, Ubolyam S, Chaiwatanarat T, Nakavachara P, Maleesatharn A, Chokephaibulkit K: **Prevalence and risk factors of low bone mineral density among perinatally HIV-infected Thai adolescents receiving antiretroviral therapy**. *J Acquir Immune Defic Syndr* 2012, **61**(4):477-483.
13. Matovu FK, Wattanachanya L, Beksinska M, Pettifor JM, Ruxrungtham K: **Bone health and HIV in resource-limited settings: a scoping review**. *Curr Opin HIV AIDS* 2016, **11**(3):306-325.
14. Slogrove AL, Schomaker M, Davies MA, Williams P, Balkan S, Ben-Farhat J, Calles N, Chokephaibulkit K, Duff C, Eboua TF *et al*: **The epidemiology of adolescents living with perinatally acquired HIV: A cross-region global cohort analysis**. *PLoS Med* 2018, **15**(3):e1002514.
15. Casado JL, Bañon S, Andrés R, Perez-Elías MJ, Moreno A, Moreno S: **Prevalence of causes of secondary osteoporosis and contribution to lower bone mineral density in HIV-infected patients**. *Osteoporosis International* 2014, **25**(3):1071-1079.
16. Weitzmann MN: **The Role of Inflammatory Cytokines, the RANKL/OPG Axis, and the Immunoskeletal Interface in Physiological Bone Turnover and Osteoporosis**. *Scientifica (Cairo)* 2013, **2013**:125705.
17. Aurpibul L, Cressey TR, Sricharoenchai S, Wittawatmongkol O, Sirisanthana V, Phongsamart W, Sudjaritruk T, Chokephaibulkit K: **Efficacy, safety and pharmacokinetics of tenofovir disoproxil fumarate in virologic-suppressed HIV-infected children using weight-band dosing.[Erratum appears in Pediatr Infect Dis J. 2015 Aug;34(8):847]**. *Pediatric Infectious Disease Journal* 2015, **34**(4):392-397.
18. Grant PM, Cotter AG: **Tenofovir and bone health**. *Current opinion in HIV and AIDS* 2016, **11**(3):326-332.
19. Hansen AB, Obel N, Nielsen H, Pedersen C, Gerstoft J: **Bone mineral density changes in protease inhibitor-sparing vs. nucleoside reverse transcriptase inhibitor-sparing highly active antiretroviral therapy: data from a randomized trial**. *HIV Med* 2011, **12**(3):157-165.
20. McComsey GA, Tebas P, Shane E, Yin MT, Overton ET, Huang JS, Aldrovandi GM, Cardoso SW, Santana JL, Brown TT: **Bone disease in HIV infection: a practical review and recommendations for HIV care providers**. *Clin Infect Dis* 2010, **51**(8):937-946.
21. Sudjaritruk T, Bunupuradah T, Aurpibul L, Kosalaraksa P, Kurniati N, Sophonphan J, Ananworanich J, Puthanakit T, Bone Dsg: **Impact of tenofovir disoproxil fumarate on bone

For peer review only - http://bmjopen.bmj.com/site/about/guidelines.xhtml

metabolism and bone mass among perinatally HIV-infected Asian adolescents. *Antiviral Therapy* 2016, **27**:27.
22. Mora S, Maruca K, Ambrosi A, Puzzovio M, Erba P, Nannini P, Benincaso A, Capelli S, Giacomet V: **Bone density, HIV infection and antiretroviral treatment: A 10-year follow-up in young patients**. *Hormone Research in Paediatrics* 2015, **84**:163-164.
23. Purdy JB, Gafni RI, Reynolds JC, Zeichner S, Hazra R: **Decreased bone mineral density with off-label use of tenofovir in children and adolescents infected with human immunodeficiency virus**. *J Pediatr* 2008, **152**(4):582-584.
24. Vancampfort D, Stubbs B, Mugisha J: **Physical activity and HIV in sub-Saharan Africa: a systematic review of correlates and levels**. *African health sciences* 2018, **18**(2):394-406.
25. Santos L, Elliott-Sale KJ, Sale C: **Exercise and bone health across the lifespan**. *Biogerontology* 2017, **18**(6):931-946.
26. Santos WR, Santos WR, Paes PP, Ferreira-Silva IA, Santos AP, Vercese N, Machado DR, de Paula FJ, Donadi EA, Navarro AM *et al*: **Impact of Strength Training on Bone Mineral Density in Patients Infected With HIV Exhibiting Lipodystrophy**. *J Strength Cond Res* 2015, **29**(12):3466-3471.
27. Dodds RM, Syddall HE, Cooper R, Kuh D, Cooper C, Sayer AA: **Global variation in grip strength: a systematic review and meta-analysis of normative data**. *Age Ageing* 2016, **45**(2):209-216.
28. Orsso CE, Tibaes JRB, Oliveira CLP, Rubin DA, Field CJ, Heymsfield SB, Prado CM, Haqq AM: **Low muscle mass and strength in pediatrics patients: Why should we care?** *Clinical Nutrition* 2019.
29. Macdonald E, Nettlefold L, Maan EJ, Cote H, Alimenti A: **Muscle power in children, youth and young adults who acquired HIV perinatally**. *J Musculoskelet Neuronal Interact* 2017, **17**(2):27-37.
30. Williams PL, Abzug MJ, Jacobson DL, Wang J, Van Dyke RB, Hazra R, Patel K, Dimeglio LA, McFarland EJ, Silio M *et al*: **Pubertal onset in children with perinatal HIV infection in the era of combination antiretroviral treatment**. *AIDS* 2013, **27**(12):1959-1970.
31. Szubert AJ, Musiime V, Bwakura-Dangarembizi M, Nahirya-Ntege P, Kekitiinwa A, Gibb DM, Nathoo K, Prendergast AJ, Walker AS, Team AT: **Pubertal development in HIV-infected African children on first-line antiretroviral therapy**. *AIDS (London, England)* 2015, **29**(5):609-618.
32. Kindblom JM, Lorentzon M, Norjavaara E, Hellqvist A, Nilsson S, Mellstrom D, Ohlsson C: **Pubertal timing predicts previous fractures and BMD in young adult men: the GOOD study**. *J Bone Miner Res* 2006, **21**(5):790-795.
33. Cousminer DL, Mitchell JA, Chesi A, Roy SM, Kalkwarf HJ, Lappe JM, Gilsanz V, Oberfield SE, Shepherd JA, Kelly A *et al*: **Genetically Determined Later Puberty Impacts Lowered Bone Mineral Density in Childhood and Adulthood**. *J Bone Miner Res* 2018, **33**(3):430-436.
34. Creo AL, Schwenk WF, 2nd: **Bone Age: A Handy Tool for Pediatric Providers**. *Pediatrics* 2017, **140**(6).
35. Crabtree N, Ward K: **Bone Densitometry: Current Status and Future Perspective**. In: *Calcium and Bone Disorders in Children and Adolescents. Volume Vol 28* 2nd, revised edition. edn. Edited by Allgrove J, Shaw NJ. Basel: Karger; 2015: pp 72-83.
36. Crabtree NJ, Shaw NJ, Bishop NJ, Adams JE, Mughal MZ, Arundel P, Fewtrell MS, Ahmed SF, Treadgold LA, Hogler W *et al*: **Amalgamated Reference Data for Size-Adjusted Bone Densitometry Measurements in 3598 Children and Young Adults-the ALPHABET Study**. *J Bone Miner Res* 2017, **32**(1):172-180.
37. Dennison EM, Jameson KA, Edwards MH, Denison HJ, Aihie Sayer A, Cooper C: **Peripheral quantitative computed tomography measures are associated with adult fracture risk: The Hertfordshire Cohort Study**. *Bone* 2014, **64**:13-17.
38. Stagi S, Cavalli L, Cavalli T, de Martino M, Brandi ML: **Peripheral quantitative computed tomography (pQCT) for the assessment of bone strength in most of bone affecting conditions in developmental age: a review**. *Italian journal of pediatrics* 2016, **42**(1):88-88.

For peer review only - http://bmjopen.bmj.com/site/about/guidelines.xhtml

39. Yin MT, Lund E, Shah J, Zhang CA, Foca M, Neu N, Nishiyama KK, Zhou B, Guo XE, Nelson J *et al*: **Lower peak bone mass and abnormal trabecular and cortical microarchitecture in young men infected with HIV early in life**. *AIDS* 2014, **28**(3):345-353.
40. **Parirenyatwa Group of Hospitals: https://parihosp.org**
41. Harare Central Hospital: **http://www.hararehospital.gov.zw**. 2019.
42. Government of Zimbabwe: **Harare Provincial Profile**. In. Harare: Parliament; 2011.
43. Rukuni R, McHugh G, Majonga E, Kranzer K, Mujuru H, Munyati S, Nathoo K, Gregson CL, Kuper H, Ferrand RA: **Disability, social functioning and school inclusion among older children and adolescents living with HIV in Zimbabwe**. *Tropical Medicine and International Health* 2017.
44. Simms V, Dauya E, Dakshina S, Bandason T, McHugh G, Munyati S, Chonzi P, Kranzer K, Ncube G, Masimirembwa C *et al*: **Community burden of undiagnosed HIV infection among adolescents in Zimbabwe following primary healthcare-based provider-initiated HIV testing and counselling: A cross-sectional survey**. *PLOS Medicine* 2017, **14**(7):e1002360.
45. Craig CL, Marshall AL, Sjöström M, Bauman AE, Booth ML, Ainsworth BE, Pratt M, Ekelund U, Yngve A, Sallis JF *et al*: **International physical activity questionnaire: 12-country reliability and validity**. *Med Sci Sports Exerc* 2003, **35**(8):1381-1395.
46. Filteau S, Rehman AM, Yousafzai A, Chugh R, Kaur M, Sachdev HPS, Trilok-Kumar G: **Associations of vitamin D status, bone health and anthropometry, with gross motor development and performance of school-aged Indian children who were born at term with low birth weight**. *BMJ Open* 2016, **6**(1).
47. FANTA: **Developing and Validating Simple Indicators of Dietary Quality and Energy Intake of Infants and Young Children in Developing Countries: Summary of findings from analysis of 10 data sets**. *Food and Nutrition Technical Assistance Project - Working Group on Infant and Young Child Feeding Indicators* 2006.
48. Foster HE, Jandial S: **pGALS - paediatric Gait Arms Legs and Spine: a simple examination of the musculoskeletal system**. *Pediatr Rheumatol Online J* 2013, **11**(1):44.
49. Crespi CM, Alfonso VH, Whaley SE, Wang MC: **Validity of child anthropometric measurements in the Special Supplemental Nutrition Program for Women, Infants, and Children**. *Pediatric research* 2012, **71**(3):286-292.
50. Baird J WI, Smith C, Inskip H. : **Review of methods for determining pubertal status and age of onset of puberty in cohort and longitudinal studies**. In: *Review of methods for determining pubertal status and age of onset of puberty in cohort and longitudinal studies.* Edited by CLOSER. London, UK: CLOSER: MRC Lifecourse Epidemiology Unit, University of Southampton; 2017.
51. Abitbol L, Zborovski S, Palmert MR: **Evaluation of delayed puberty: what diagnostic tests should be performed in the seemingly otherwise well adolescent?** *Archives of Disease in Childhood* 2016, **101**:767-771.
52. Martin DD, Wit JM, Hochberg Z, Sävendahl L, van Rijn RR, Fricke O, Cameron N, Caliebe J, Hertel T, Kiepe D *et al*: **The Use of Bone Age in Clinical Practice – Part 1**. *Hormone Research in Paediatrics* 2011, **76**(1):1-9.
53. Biver E, Calmy A, Delhumeau C, Durosier C, Zawadynski S, Rizzoli R: **Microstructural alterations of trabecular and cortical bone in long-term HIV-infected elderly men on successful antiretroviral therapy**. *AIDS* 2014, **28**(16):2417-2427.
54. Clark EM, Ness AR, Tobias JH: **Bone fragility contributes to the risk of fracture in children, even after moderate and severe trauma**. *J Bone Miner Res* 2008, **23**(2):173-179.
55. Washington Group on Disability Statistics, UNICEF: **Module on Child Functioning and Disability** *Available online from http://wwwwashingtongroup-disabilitycom/wp-content/uploads/2016/02/wg_unicef_child-disability-background-documentpdf* 2014.
56. Marshall WA, Tanner JM: **Variations in pattern of pubertal changes in girls**. *Arch Dis Child* 1969, **44**(235):291-303.
57. Marshall WA, Tanner JM: **Variations in the pattern of pubertal changes in boys**. *Arch Dis Child* 1970, **45**(239):13-23.

For peer review only - http://bmjopen.bmj.com/site/about/guidelines.xhtml

58. **The WHO child growth standards.Growth reference, 5–19y.** [Geneva, Switzerland: World HealthOrganization; 2007 http://www.who.int/childgrowthref/en/13]
59. Häger-Ross C, Rösblad B: **Norms for grip strength in children aged 4-16 years**. *Acta Paediatr* 2002, **91**(6):617-625.
60. Armstrong M: **Youth Fitness Testing in South African Primary School Children: National Normative Data, Fitness and Fatness, and Effects of Socioeconomic Status**. Cape Town: University of Cape Town; 2009.

For peer review only

For peer review only - http://bmjopen.bmj.com/site/about/guidelines.xhtml

TABLES AND FIGURES

Table 1. Summary of study measurements to be quantified at baseline and follow-up

	Measurement	Measurement method	Outcome
INTERVIEW BASED QUESTIONNAIRE	Socio-demographic characteristics	Questionnaire	Age, sex, school attendance, orphanhood, guardianship
	Clinical history	Questionnaire[a]	History of fractures and trauma (modified Landin classification [54])<br>*HIV history: age at diagnosis, WHO disease stage, nadir CD4 count, opportunistic infections<br>*ART regimen/duration,<br>Exposures: steroid use, smoking, alcohol, recreational drugs<br>Family history of musculoskeletal disease & fractures<br>Other co-morbidities
	Physical activity	The International Physical Activity Questionnaire (IPAQ) [45] questionnaire (short form)	Median MET-minutes[b] of physical activity/week<br>1. inactive (<600 MET-minutes/week)<br>2. minimally active (600-1499 MET-minutes/week)<br>3. highly active (≥1500 MET-minutes/week)
	Nutrition[b]	Dietary assessment tool (Modified short food frequency questionnaire [46])	Daily dietary calcium and vitamin D intake<br>Prevalence of vitamin supplementation<br>Sun exposure
	Quality of life and disability	Washington Disability Score [55]	Functioning and disability score
STANDARDISED EXAMINATION	Musculoskeletal examination	Paediatric Gait Arms Legs and Spine (pGALS)[48] +/- regional clinical examination	Joint, spine and gait abnormalities
	Pubertal stage	Tanner's staging [56, 57]	Pre-pubertal (Stage 1)<br>Pubertal (Stage 2-3)<br>Post-pubertal (Stage 4 & 5)
	Anthropometry	Height (standing & sitting)<br>Weight<br>Mid-upper arm circumference (MUAC)[c]	Standing height-for-age (Z-score) [58][d]<br>Weight-for-age (Z-score) [58][d]<br>Body Mass Index (BMI) (Z-score) [58][d]<br>MUAC (Z-score) [58][d]
	Muscle strength	Jamar Dynamometer<br>Standing long jump[d]	Hand grip strength (kg, Z-score) [59][d]<br>Jumping distance (cm, Z-score) [60][e]
RADIOLOGY	Skeletal maturity	Hand/ wrist radiograph	Bone age (years)
	Bone and muscle composition	Dual-energy X-ray absorptiometry (DXA) of total body, lumbar spine and hip	Size corrected DXA measures of TBLH BMC^{LBM} (g), LS BMAD (g/cm^3) and Z-scores <-2.[d]<br>Lean mass
	Bone architecture	Peripheral quantitative computed tomography (pQCT)	Trabecular and cortical vBMD (g/cm^3),<br>Total and cortical CSA (mm^2), cortical thickness (mm), Periosteal and endosteal circumference (mm), SSI (mm^3) PMI (mm^4) and CSMI (mm^4)
BLOOD TESTS	Bone markers and DNA	Blood test (DNA extraction and serum saved)	Future testing
	HIV markers	Blood test	*CD4 count, HIV viral load

For peer review only - http://bmjopen.bmj.com/site/about/guidelines.xhtml

Table 1. Footnotes

a) Details of treatment and co-morbidities will be confirmed by patient-held medical records where available. **b)** Energy requirements defined in METS (multiples of the resting metabolic rate that give a score in MET-minutes). **c)** Nutritional indicator to include composite information from history (usual diet last month, sun exposure- vitamin D status) and clinical exam (MUAC). Similar methods have been used in other low income contexts [46]. **d)** Age and sex specific Z-scores for 1) *anthropometric measures:* will be determined using WHO child growth standards [58]; 2) hand *grip strength*: will be determined with reference to the uninfected comparison group and European normative data [59]; 3) *jumping distance:* will be determined using normative data from South Africa [60] 4) *low BMD* will be determined with reference to published paediatric Hologic DXA reference databases for LS BMAD and TBLH BMC^{LBM} Z-scores [36]. **e)** Standing long jump; the longest distance after two attempts will be recorded. **f)** Pregnancy urine dipstick in females prior to DXA if uncertain pregnancy status. **g)** Tests to be carried out on stored blood when further funding is secured.

*Denotes assessments to be carried out in HIV-infected participants only. **Abbreviations:** CSA (cross-sectional area), CSMI (cross sectional moment of inertia), LS BMAD (lumbar spine bone mineral apparent density) PMI (polar moment of inertia), SSI (Strength Strain Index), TBLH BMC^{LBM} (total-body less-head bone mineral content for lean mass adjusted for height).

Figure 1. Hypothesized changes in bone mass across the life-course in HIV-infected and uninfected individuals

Figure 2. Hypothesised growth scenarios to be assessed as interactions between pubertal stage and HIV status on change in bone mass

For peer review only

For peer review only - http://bmjopen.bmj.com/site/about/guidelines.xhtml

Figure 1. Hypothesized changes in bone mass across the life-course in HIV-infected and uninfected individuals

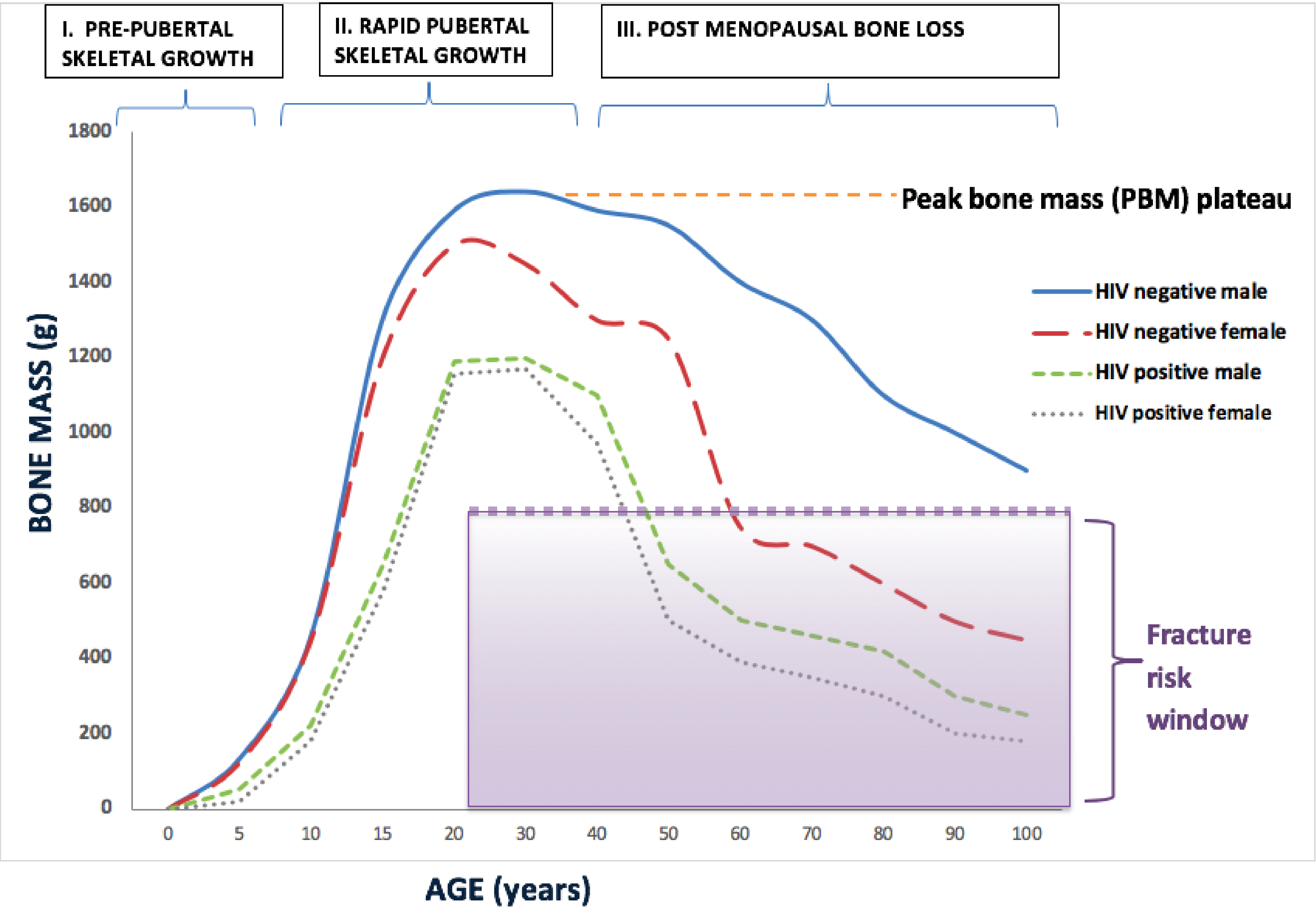

Figure 2. Hypothesised growth scenarios to be assessed as interactions between pubertal stage and HIV status on change in bone mass

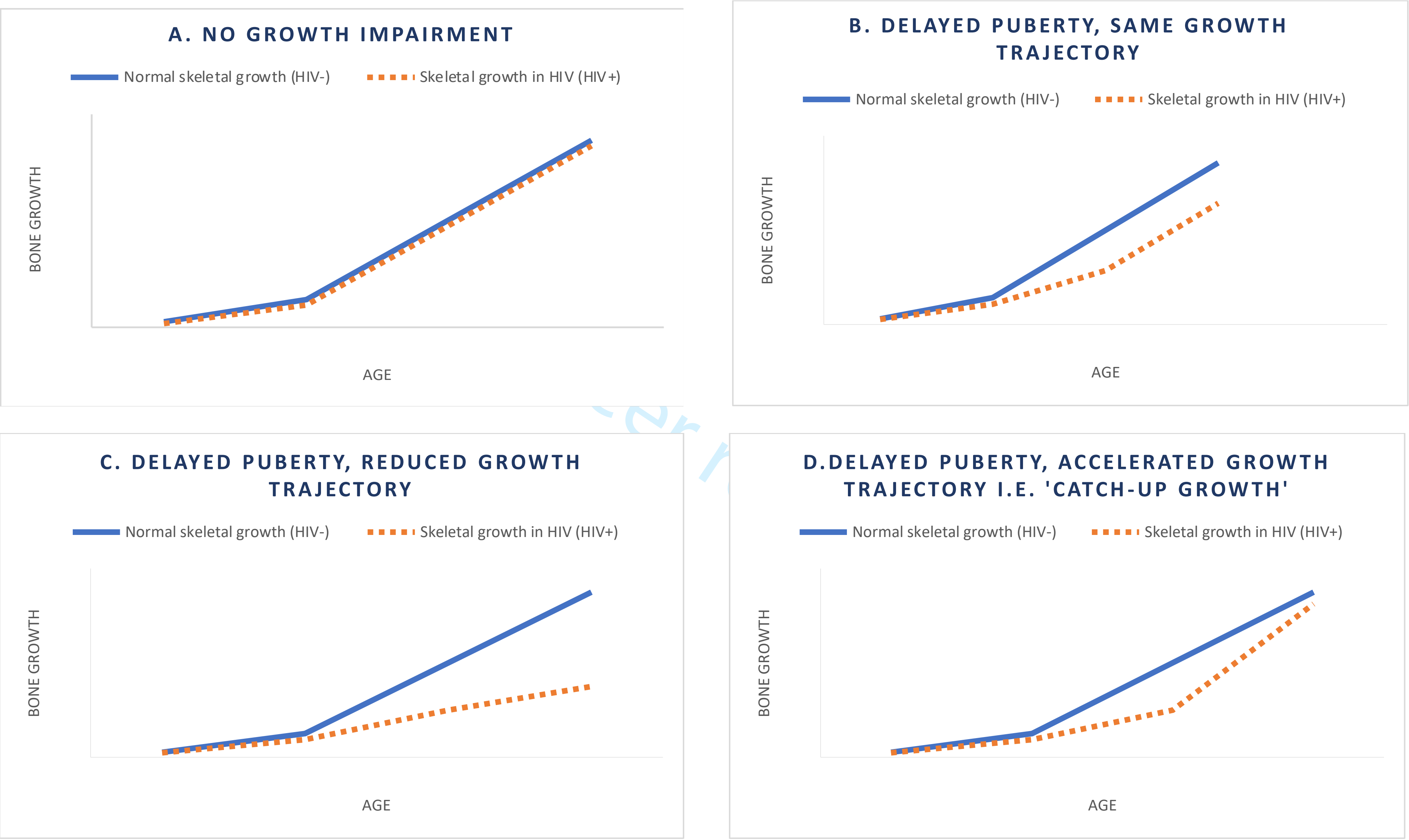

BMJ Open

The IMpact of Vertical HIV infection on child and Adolescent Skeletal development in Harare, Zimbabwe (IMVASK Study): a protocol for a prospective cohort study

Journal:	*BMJ Open*
Manuscript ID	bmjopen-2019-031792.R2
Article Type:	Protocol
Date Submitted by the Author:	08-Dec-2019
Complete List of Authors:	Rukuni, Ruramayi; London School of Hygiene and Tropical Medicine, Clinical Research Department; Biomedical Research and Training Institute, Harare<br>Gregson, Celia; University of Bristol, Musculoskeletal Research Unit; Royal United Hospital NHS Trust, Older Person's Unit<br>Kahari, Cynthia; London School of Hygiene and Tropical Medicine, 4. Department of Infectious Disease Epidemiology, Faculty of Epidemiology and Population Health; Biomedical Research and Training Institute (BRTI)<br>Kowo, Farirayi; University of Zimbabwe, Department of Radiology<br>McHugh, Grace; Biomedical Research and Training Institute, Harare<br>Munyati, Shungu; Biomedical Research and Training Institute, Harare<br>Mujuru, Hilda; University of Zimbabwe , College of Health Sciences<br>Ward, Kate; MRC Lifecourse Epidemiology Unit<br>Filteau, Suzanne; London School of Hygiene & Tropical Medicine, Population Health<br>Rehman, Andrea; London School of Hygiene and Tropical Medicine, Infectious Disease Epidemiology<br>Ferrand, Rashida; London School of Hygiene and Tropical Medicine
**Primary Subject Heading**:	Public health
Secondary Subject Heading:	Paediatrics, Radiology and imaging
Keywords:	HIV & AIDS < INFECTIOUS DISEASES, Tropical medicine < INFECTIOUS DISEASES, Paediatric radiology < PAEDIATRICS, Epidemiology < TROPICAL MEDICINE

SCHOLARONE™
Manuscripts

I, the Submitting Author has the right to grant and does grant on behalf of all authors of the Work (as defined in the below author licence), an exclusive licence and/or a non-exclusive licence for contributions from authors who are: i) UK Crown employees; ii) where BMJ has agreed a CC-BY licence shall apply, and/or iii) in accordance with the terms applicable for US Federal Government officers or employees acting as part of their official duties; on a worldwide, perpetual, irrevocable, royalty-free basis to BMJ Publishing Group Ltd ("BMJ") its licensees and where the relevant Journal is co-owned by BMJ to the co-owners of the Journal, to publish the Work in this journal and any other BMJ products and to exploit all rights, as set out in our licence.

The Submitting Author accepts and understands that any supply made under these terms is made by BMJ to the Submitting Author unless you are acting as an employee on behalf of your employer or a postgraduate student of an affiliated institution which is paying any applicable article publishing charge ("APC") for Open Access articles. Where the Submitting Author wishes to make the Work available on an Open Access basis (and intends to pay the relevant APC), the terms of reuse of such Open Access shall be governed by a Creative Commons licence – details of these licences and which Creative Commons licence will apply to this Work are set out in our licence referred to above.

Other than as permitted in any relevant BMJ Author's Self Archiving Policies, I confirm this Work has not been accepted for publication elsewhere, is not being considered for publication elsewhere and does not duplicate material already published. I confirm all authors consent to publication of this Work and authorise the granting of this licence.

For peer review only

The IMpact of Vertical HIV infection on child and Adolescent Skeletal development in Harare, Zimbabwe (IMVASK Study): a protocol for a prospective cohort study

Ruramayi Rukuni[1,2], Celia L Gregson[3], Cynthia Kahari[2,4], Farirayi Kowo[5], Grace McHugh[2], Shungu Munyati[2], Hilda Mujuru[6], Kate A Ward[7], Suzanne Filteau[8], Andrea M Rehman[4] and Rashida A Ferrand[1,2]

Affiliations:

1. Clinical Research Department, Faculty of Infectious and Tropical Diseases, London School of Hygiene and Tropical Medicine (LSHTM), London, UK.
2. Biomedical Research and Training Institute (BRTI), Harare, Zimbabwe.
3. The Musculoskeletal Research Unit, Translational Health Sciences, Bristol Medical School, University of Bristol, Bristol, UK.
4. Department of Infectious Disease Epidemiology, Faculty of Epidemiology and Population Health, London School of Hygiene and Tropical Medicine (LSHTM), London, UK.
5. Department of Radiology, University of Zimbabwe, Harare, Zimbabwe.
6. Department of Paediatrics, University of Zimbabwe, Harare, Zimbabwe.
7. MRC Lifecourse Epidemiology Unit, University of Southampton, Southampton, UK.
8. Department of Population Health, Faculty of Epidemiology and Population Health, London School of Hygiene and Tropical Medicine (LSHTM), London, UK.

Corresponding Author

Dr Ruramayi Rukuni

Biomedical Research and Training Institute (BRTI), 10 Seagrave Rd, Avondale, Harare, Zimbabwe.

Tel: +263 719 362 961

Email: Ruramayi.Rukuni@lshtm.ac.uk

For peer review only - http://bmjopen.bmj.com/site/about/guidelines.xhtml

ABSTRACT

Introduction

The scale-up of antiretroviral therapy (ART) across sub-Saharan Africa (SSA) has reduced mortality so that increasing numbers of children with HIV (CWH) are surviving to adolescence. However, they experience a range of morbidities due to chronic HIV infection and its treatment. Impaired linear growth (stunting), is a common manifestation, affecting up to 50% of children. However, the effect of HIV on bone and muscle development during adolescent growth is not well characterised. Given the close link between pubertal timing and musculoskeletal development, any impairments in adolescence are likely to impact on future adult musculoskeletal health. We hypothesize that bone and muscle mass accrual in CWH is reduced, putting them at risk of reduced bone mineral density (BMD) and muscle function and increasing fracture risk. This study aims to determine the impact of HIV on BMD and muscle function in peri-pubertal children on ART in Zimbabwe.

Methods and analysis

CWH (n=300) and without HIV (n=300), aged 8-16 years, established on ART, will be recruited into a frequency-matched prospective cohort study and compared. Musculoskeletal assessments including dual-energy X-ray absorptiometry (DXA), peripheral quantitative computed tomography (pQCT), grip strength and standing long jump will be conducted at baseline and after one year. Linear regression will be used to estimate mean size-adjusted bone density and Z-scores by HIV status (*i.e.* total-body less-head (TBLH) bone mineral content (BMC) for lean mass adjusted for height (TBLH BMC^{LBM}) and lumbar spine bone mineral apparent density (LS BMAD). The prevalence of low size-adjusted BMD (i.e. Z-scores <-2) will also be determined.

Ethics and dissemination

Ethical approval for this study has been granted by the Medical Research Council of Zimbabwe and the LSHTM Ethics Committee. Baseline and longitudinal analyses will be published in peer reviewed journals and disseminated to research communities.

For peer review only

For peer review only - http://bmjopen.bmj.com/site/about/guidelines.xhtml

STRENGTHS AND LIMITATIONS OF THIS STUDY

- This study will provide novel understanding of the effects of HIV on bone and muscle development in a large population of sub-Saharan African (SSA) children living with HIV by using 'gold standard' size adjustment methods for DXA, which are crucial for assessing a population with inherent size differences
- Bone architecture measurement using pQCT will provide understanding of trabecular and cortical bone geometry and strength in CWH
- This study will generate new data for total body and lumbar spine DXA, tibial pQCT, hand grip strength and standing long jump for Zimbabwean children without HIV which will inform normative reference data
- Whilst the age range in this study, 8-16 years, will allow analysis of pubertal delay in children with HIV, the follow-up period is insufficient to determine the impact on attainment of peak bone mass which probably occurs in the early twenties.

INTRODUCTION

Sub-Saharan Africa (SSA) disproportionally bears the burden of global HIV infection, with nearly 90% of the estimated 2.1 million children under 15 years of age living in SSA [1]. The global scale-up of antiretroviral therapy (ART) has dramatically improved survival of children with HIV (CWH) [2]. However there is accumulating evidence that the growing number of these children are now reaching adolescence in SSA with multisystem chronic comorbidities associated with HIV infection and/or its treatment [3].

Poor linear growth (*i.e*. stunting), is one of the most common manifestations of perinatally (vertically)-acquired HIV infection, affecting up to 50% of children [4, 5]. Linear growth is greatest in adolescence during the pubertal development period. Bone mass is thought to change throughout the life course and may be altered by HIV [6, 7] (Figure 1). The majority of peak bone mass (PBM), the maximum amount of bone accrued by the end of skeletal maturation, is attained during adolescent growth; by age 18 years in women and age 20 years in men, 80% of PBM is attained [8]. After PBM is reached, there is no net gain in bone mass. Therefore PBM is the net reservoir of bone for later life, a key determinant of adult bone mineral density (BMD) and consequently of adult osteoporotic fracture risk [9]. Linear growth is therefore intimately linked to skeletal development but how HIV infection affects bone development in peri-pubertal SSA children is largely unknown. The prevalence of low BMD has been found to be higher in CWH than uninfected children in high and middle income countries (7% in

For peer review only - http://bmjopen.bmj.com/site/about/guidelines.xhtml

the USA [10], 32% in Brazil [11] and 24% in Thailand [12] compared to 1% in children without HIV in the USA [10]. No study has estimated the prevalence of low BMD in SSA, and the prevalence of and risk factors for low BMD in African CWH is not known [6, 13]. It is important to highlight that the risk of poor bone accrual, reflected in low BMD measurements, is likely to be different in low income countries compared to high income countries due to factors such as malnutrition and social deprivation; but critically due to delayed ART initiation. A recent meta-analysis has shown that the median age of ART initiation in the UK/USA is two years, compared to eight years in SSA [14].

The mechanisms by which HIV may lead to low size-adjusted BMD in children are not fully understood but are likely multifactorial including HIV-associated factors (*e.g.* ART drugs, HIV disease stage) and traditional risk factors (*e.g.* hypogonadism, smoking, alcohol, low physical activity and vitamin D deficiency) [15]. HIV infection promotes systemic immune activation and production of inflammatory cytokines (*e.g.* TNFα) that in turn promote increased bone resorption [16]. ART initiation, particularly with tenofovir (part of the first-line ART regimen in SSA), predicts an initial decline in BMD which stabilizes after two years in adults [17]. It is thought tenofovir may cause renal proximal tubule toxicity resulting in phosphate wasting and increased bone turnover [18]. Although tenofovir and protease inhibitors have been associated with low BMD in adults [19, 20], studies in children have shown inconsistent findings [21-23]. Malnutrition, opportunistic infections and social deprivation may also impede musculoskeletal development. Reduced physical activity, associated with HIV [24], may also impair muscle development and limit impact loading to reduce osteocyte-mediated bone accrual [25, 26] . In adults, weak grip strength has been associated with increased falls and fracture risk [27]. Although muscle (lean) mass has also been shown to predict the magnitude of bone accrual during growth [28], few studies have compared muscle strength and function between children with and without HIV. Interestingly, a small Canadian study showed deficits in muscle power in CWH [29].

Another mechanism by which HIV may exert effects on BMD is through its effect on puberty. Even in the presence of ART, the onset of puberty is delayed by approximately a year in CWH in both high income [30] and low income settings [31]. Older age at ART initiation has been shown to be a significant risk factor for pubertal delay in Zimbabwean CWH [31]. Pubertal delay in HIV may be mediated through nutritional deficiency, recurrent infection, or chronic immune activation disrupting hormonal regulation [31]. Delayed puberty may be advantageous for linear growth; spending more time in puberty may allow more time for skeletal growth [31]. Conversely, delayed puberty has been shown in studies in high income settings to be detrimental to bone mass accrual [32, 33]. However, the impact of pubertal delay on BMD in low income countries remains unknown. Pubertal delay can be assessed objectively using hand radiographs. Analysis of the growth plate development and fusion

For peer review only - http://bmjopen.bmj.com/site/about/guidelines.xhtml

of long bones in the hands can accurately quantify bone age, which is a measure of skeletal maturation. Bone age lagging behind chronological age reflects pubertal delay [34].

BMD is commonly measured by Dual-energy X-ray absorptiometry (DXA) as two-dimensional (areal) BMD, however, this is highly dependent on bone size [35]. DXA underestimates areal bone density in short children, with smaller bones, and overestimates BMD in taller children, with bigger bones, despite the fact that they may have identical volumetric BMD. Size adjustment of DXA measures is therefore critically important in children with chronic diseases such as HIV, where smaller size due to poorer growth and delayed puberty may explain findings of lower BMD. The two 'gold standard' size-adjustment techniques chosen from the International Society for Clinical Densitometry (ISCD) are: bone mineral apparent density at the lumbar spine (LS BMAD) and regression based total-body less-head (TBLH) Bone Mineral Content (BMC) for lean mass adjusted for height (TBLH BMC^{LBM}) [36] Z-scores. As there are currently no published reference DXA data for child or adolescent populations in SSA, in this study we will use the of best available data sets from high income countries such as the UK [36] to generate Z-scores.

Unlike DXA, peripheral quantitative computed tomography (pQCT) takes into account bone size by directly measuring volumetric BMD. It has the additional advantage of separately assessing trabecular and cortical bone compartments, providing information on bone architecture. Furthermore, a range of bone strength indices *e.g.* strength stain index, validated against fracture risk can be calculated [37, 38]. In high income countries, markedly abnormal trabecular and cortical architecture have been shown in adults with HIV [39] and abnormal bone architecture and impaired bone strength through to early adulthood have been shown in boys with HIV infection [39]. Few studies have assessed bone architecture and strength in CWH in SSA.

The IMVASK study aims to determine the prevalence of low size-adjusted BMD and muscle function (grip strength and standing long jump) in Zimbabwean children with and without HIV. pQCT assessment will enable understanding of the impact of HIV infection on bone architecture and strength. This study will further contribute to local reference data for DXA measures, bone age and muscle function (grip strength and standing long jump) for a sub-Saharan African population, establishing a biorepository for future research. Study results will aid understanding of bone and muscle accrual in the context of HIV infection in the era of ART.

For peer review only - http://bmjopen.bmj.com/site/about/guidelines.xhtml

METHODS AND ANALYSIS

Study objectives

To determine the impact of HIV infection on size-adjusted bone density in peri-pubertal children aged 8-16 years established on ART. The objectives of this prospective study are:

1) To quantify the prevalence of low size-adjusted BMD and low muscle function (grip strength and standing long jump) among CWH compared to uninfected children
2) To investigate the risk factors for low size-adjusted bone density and low muscle function (grip strength and standing long jump) among children with HIV
3) To compare the rates of bone mass accrual over one year between children with and without HIV and assess for interaction by pubertal stage to determine if CWH exhibit catch up growth
4) To determine the differences in bone architecture measured by pQCT between children with and without HIV

Study hypothesis

We hypothesize that HIV infection adversely affects skeletal development, such that CWH, despite ART, accrue less bone mass and strength and have reduced muscle function during skeletal development.

Study design

CWH aged 8-16 years and established on ART (n=300) and a comparison group of children without HIV, frequency-matched for age and sex (n=300) will be recruited into a prospective cohort study. Detailed musculoskeletal assessments will be conducted at baseline and after one year.

Study setting

Parirenyatwa and Harare Hospital are the largest public-sector referral hospitals in Harare [40, 41]. The paediatric HIV clinics at both hospitals provide HIV care to more than 2,000 children. Although HIV care is increasingly decentralised to primary care level across the country, most children in Harare continue to receive care within HIV clinics in secondary healthcare facilities. Parirenyatwa hospital has a well-functioning radiology department which houses the University of Zimbabwe DXA and pQCT research unit and has access to private radiology services in the surrounding area. The hospital catchment areas have over 116 primary and 42 secondary government schools with an estimated 157,962 children enrolled [42]. School attendance in Harare province is high and does not differ by HIV status, with 96% of children under 18 years attending school [43].

For peer review only - http://bmjopen.bmj.com/site/about/guidelines.xhtml

Recruitment of participants

Eligibility

Inclusion criteria: age 8-16 years (includes pre- and peri-pubertal children), living in Harare, and in CWH only if:

i. taking ART for at least two years (as adult studies demonstrate ART initiation is followed by an initial decline in BMD which stabilizes after 2 years [17]).
ii. the child is aware of their HIV status, to avoid inadvertent disclosure as a result of study participation.

Children with perinatally acquired HIV will be included in this study. Perinatally-acquired HIV will be defined based on Zimbabwean criteria *i.e.* self-report of no sexual debut or blood transfusions, a history of natural sibling or maternal HIV and characteristic clinical features of longstanding HIV. Children with horizontal infection will also be included in the study.

Exclusion criteria: acute illness (requiring immediate hospitalisation) and lack of consent.

Detailed information on all the above comorbidities will be collected using the main study questionnaire in the clinical history section. This information will be collected for both children with and without HIV. Co-morbidities will not be used as the basis of excluding children from the study. However, for the purposes of deriving normative DXA data, those with severe bone disease will be excluded at the analysis stage.

Recruitment of children with HIV

Systematic quota-based sampling by age and sex will be used to recruit 300 children from Parirenyatwa and Harare Hospital HIV clinics. Participants will be recruited sequentially as they attend clinic such that 50 males and 50 females will be chosen for each of three age-strata, 8-10.99, 11-13.99 and 14-16.99 years. A maximum of 5 participants will be enrolled on each day for logistical reasons. The total number of children approached each day will be recorded, irrespective of whether they are subsequently eligible or enrolled to determine the sampling fraction. Written consent will be obtained from children and their guardians. Study processes and procedures will be clearly explained to children and their guardians and they will be given the option to accept or decline to take part in the research. It will also be explained that they are allowed to withdraw from the study at any time, for any reason, without affecting the care they receive from the clinic.

Recruitment of children without HIV

Three hundred CWH will be randomly sampled from six government primary and secondary schools in the same catchment area as Parirenyatwa and Harare Hospitals. Younger children (8-12 years) will be selected from primary schools and older children (13-16 years) from secondary schools, with

For peer review only - http://bmjopen.bmj.com/site/about/guidelines.xhtml

thirteen-year olds coming from both primary and secondary schools. The number of children selected from each school will be proportional to school size, thereby giving each child equal probability of being sampled. A random number sequence will be generated, and school registers will be used to select participants of similar age and sex as the children with HIV using the same quota-based approach of 50 males and 50 females in each of the three age strata. Guardians of selected school children will be invited to the study clinic to complete the consent process. Consenting participants will have a diagnostic HIV test as part of their assessment. Those testing HIV positive (anticipated to be approximately 2-3% [44]) will be referred for HIV care.

Study procedures

Questionnaire

An interviewer-administered questionnaire together with hand-held medical records will be used to collect socio-demographic details and clinical history including age, sex, school attendance, orphan status, guardianship, history of fractures with mechanism of trauma, steroid use, smoking, alcohol, recreational drugs, family history of musculoskeletal disease, co-morbidities, physical activity, diet and nutrition and sun exposure. Where possible, validated instruments adapted for the local context will be used. For example, the International Physical Activity Questionnaire (IPAQ) [45] validated in multiple countries including South Africa and will be used to assess physical activity as multiples of the resting metabolic rate (MET) in MET-minutes. Diet and nutrition will be assessed using a tool we developed for the Zimbabwean context based on a validated dietary diversity and food frequency tool from India and Malawi [46] and international guidelines applicable to SSA [47]. The tool quantifies vitamin D supplementation and sunlight exposure and has been adapted to reflect the Zimbabwean context where fortification of oils and margarine with vitamin D is mandated by the government and specific vitamin D rich foods such as kapenta fish are found.

Clinical examination

A standardised musculoskeletal examination will be conducted using the validated paediatric gait, arms, legs and spine (pGALS) examination [48]. Additional clinical assessments will be carried out using standardised protocols and calibrated equipment. Anthropometry measurements will include standing and sitting height, arm span, mid upper arm circumference. Height will be measured to the nearest 0.1 cm, by two separate readers using calibrated Seca 213 stadiometers. If the two height measurements differ by more than 0.5 cm , a third reading will be taken [49]. Weight will be measured to the nearest 0.1 kg using calibrated Seca 875 scales. Tanner pubertal staging will be carried out using a standardised protocol with an orchidometer to assess testicular volume in males [50]. Muscle function will be assessed in the upper limb and lower limbs by grip strength dynamometry and

For peer review only

For peer review only - http://bmjopen.bmj.com/site/about/guidelines.xhtml

standing long jump respectively. Hand grip strength will be measured using a Jamar hydraulic hand-held dynamometer (Patterson Medical, UK) to the nearest 0.1kg. Participants will be seated with the shoulder at 0° to 10°, the elbow at 90° of flexion and the forearm positioned neutrally. Three measurements will be taken from each hand in alternation and the highest measurement chosen. The standing long jump distance will be taken from the best of three correctly performed attempts to the nearest 0.1 cm, measuring the distance from the take-off line to the heel.

Radiological assessments

DXA scans will be performed by two trained radiographers using a Hologic QDR Wi densitometer with Apex software version 4.5. Measurements will be taken from the lumbar spine, hip and total body. Fat and muscle mass will also be acquired; muscle mass is the fat free mass measurement from DXA. DXA scans will be repeated in a subgroup (n=20) of participants to determine reproducibility. pQCT measurements of the non-dominant tibia will be taken using a Stratec XCT-2000 scanner (Stratec, Pforzheim, Germany) software version 6.20. Measurements of the non-dominant tibia will be taken at three sites at 4%, 38%, and 66% percent of the tibial length, measured from the medial malleolus to the medial tibial plateau. Daily quality control will be performed by scanning the manufacturer provided lumbar spine phantom for DXA and tibia phantom for pQCT. A radiograph of the non-dominant hand and wrist will be taken and used to quantify bone age using the Greulich and Pyle (G&P) atlas and the Tanner Whitehouse 3 (TW3) method. For Intra-observer reliability, 10% of the radiographs will be randomly selected and rescored by the same operator after one week. For inter-observer reliability a different set of 10% of the radiographs will be re-scored by a different expert. The estimated bone age will then be compared to the calculated chronological age.

Blood tests

A fasting blood sample (up to 15ml) will be collected. HIV markers (CD4 count and viral load) will be tested in CWH only. CD4 cell count will be measured using an Alere PIMA CD4 machine (Waltham, Massachusetts, USA). HIV viral load will be measured using the GeneXpert HIV-1 viral load platform (Cepheid Inc, Sunnyvale, California, USA). The remaining blood plasma will be bio-banked to enable future measurement of bone biochemistry. After removing the plasma, peripheral blood mononuclear cells (PBMC) will be isolated and cryopreserved. DNA will also be extracted using a manual method and stored for future genetic studies.

Follow up at one year

All study measurements, with the exception of DNA extraction, will be repeated after one year. Participants will be recalled exactly one year after their first DXA scan. The aim is to perform all scans within a 4 week window period. Contact will be maintained with participants via regular phone calls

For peer review only - http://bmjopen.bmj.com/site/about/guidelines.xhtml

and text messaging to minimise loss-to-follow-up. The schedule of study procedures is summarised in Table 1.

Outcome measures

The primary study outcomes are:

1) mean size-adjusted bone density Z-scores; TBLH BMC^{LBM} and LS BMAD [36].
2) the prevalence of low TBLH BMC^{LBM} and LS BMAD Z-score <-2 at baseline [36].

Secondary study outcomes are:

1) prevalence of low muscle function; grip strength and standing long jump-for-age (Z-score<-2) and musculoskeletal abnormalities/disabilities by HIV status at baseline.
2) mean percentage change in TBLH BMC^{LBM} (g) and LS BMAD (g/cm^3), tibial cortical and trabecular volumetric BMD (g/cm^3), total cross sectional area, cortical thickness and bone strength, muscle mass and function at baseline and one year, by HIV status.
3) assessment of the extent to which pubertal delay explains changes in these bone and muscle outcomes.

Sample size

The sample size was calculated to detect differences in DXA-measured mean size-adjusted bone BMD Z-scores between children with and without HIV. This study will have 80% power (α 0.05) to detect a 0.23 Z-score difference between 300 HIV-infected and 300 uninfected children, assuming a standard deviation of 1.3. As there were no published studies from low income countries, estimates of the expected difference were taken from a US study of children with HIV aged 7 to 15 years [10]. In addition, our study will have 80% power to detect a 4.8% difference in the prevalence low size-adjusted BMD between the two groups. This is a smaller prevalence difference than that detected by the most conservative prevalence estimate of low BMD of 7% from three studies in high and middle-income countries [10-12].

Statistical analysis

For continuous variables that are normally distributed, the mean and standard deviation will be presented. For skewed continuous variables, the median and inter-quartile range (IQR) will be presented. Categorical variables will be summarised as frequencies and percentages. The distribution of demographic and clinical variables will be compared between CWH and without HIV using independent sample *t*-tests for means, Wilcoxon rank sum test for medians and Chi-squared tests for proportions.

For peer review only - http://bmjopen.bmj.com/site/about/guidelines.xhtml

Baseline mean TBLH BMC^{LBM} and LS BMAD Z-scores and the prevalence of low TBLH BMC^{LBM} and LS BMAD Z-score will be compared between CWH and without HIV. Among CWH, the association between *a priori* defined risk factors (ART duration, ART type, proportion of life on treatment, age at ART initiation, CD4 count, viral load, bone age, pubertal stage, nutrition, socioeconomic status and orphanhood) against size-adjusted BMD will be examined using multivariable linear regression (Z-score as a continuous variable) and multivariable logistic regression (as defined by the Z-score cut off of <-2). Successful antiretroviral therapy will be defined as a viral load of less than 1, 000 copies/ml. Paired sample t test or nonparametric Wilcoxon test will be used to assess for differences in TBLH BMC and LS BMAD on CWH and children without HIV between baseline and follow up. Multivariable linear regression will be used to analyse the mean percentage change in TBLH BMC^{LBM}(g) and LS BMAD (g/cm^3) between children with and without HIV. Models will be adjusted for physical activity, calcium and vitamin D intake. Interaction between the effects of pubertal stage (bone age) and HIV on change in TBLH BMC^{LBM} and LS BMAD will be investigated to see if differences in bone density become more or less pronounced through puberty *i.e.* whether catch-up growth is possible, see Figure 2. The regression coefficient (β) for percentage change in size-adjusted bone mass may suggest either no growth impairment (Figure 2A), delayed puberty whilst maintaining the same growth trajectory (Figure 2B) or delayed puberty with a reduced growth trajectory (Figure 2C) in CWH. If β is markedly more positive in CWH, this suggests that catch-up growth may be possible (Figure 2D). Pubertal delay in this study will be defined as the lack of the initial signs of puberty (Tanner stage 2) at an age that is more than 2 standard deviations beyond the population mean [51] and as chronological age minus bone age > 2 years [52]. Data for total body and lumbar spine DXA, tibial pQCT, hand grip strength and standing long jump in CWH will be analysed with reference to the comparator group of children without HIV.

For the purposes of normative data derivation, children without HIV who have any diagnosis or evidence of muscle or bone disease will be excluded. Then outliers with bone density, hand grip strength or standing long jump data beyond 2 standard deviations from the mean will have their case record reviewed to exclude cases with underlying bone or muscle pathology. The remaining population will be used to generate normative references ranges for these quantitative traits.

Data management

Data collection, management and storage will be governed by standard operating procedures and will follow the principles of Good Clinical Practice (GCP). Data will be captured using hand held tablets for the questionnaires. Paper forms will be available in case of failure of electronic data entry. Microsoft Access will be used as the main backend database as it allows programming of quality control checks

For peer review only - http://bmjopen.bmj.com/site/about/guidelines.xhtml

and conditional data validation. GCP compliant audit trail modules will be incorporated into the databases and reports of aggregated data will be reviewed on a monthly basis. In order to assure data quality and consistency, all staff will receive regular training and regular quality checks will be conducted. Paper records will be stored for eight years after the completion of research in secure, locked storage facilities. Field staff will download data to the central database, which is backed up onto an encrypted external hard drive daily, and to additional off-site and secure cloud back-up. The off-site back-up copies will be stored through the London School of Hygiene and Tropical Medicine (LSHTM) Research Data Management Support Service that has an established data repository. In order to preserve the long-term value of this data, it will be stored backed-up here indefinitely. Anonymised research data will be made available for sharing through the open access data repository established by the LSHTM Data Management Support Service at the time of publication. This will allow other research groups to request access to study data and tools. Information on how other researchers' data will be included in every study publication.

For peer review only

Patient and Public Involvement

Whilst patients were not directly involved in the design and conduct of the study, feedback from patient experiences in the study will be used to inform planned public engagement activities, which include science fairs, conducted by the research team at schools from where participants were recruited.

Study status

Recruitment to this study began in May 2018 and is planned until August 2019. Study follow up will run from May 2019 to August 2020.

DISCUSSION

Although the scale-up of prevention of mother-to-child transmission (PMTCT) has reduced perinatal HIV transmission but coverage is still not universal in most parts of SSA and therefore perinatal HIV infection is expected to affect large numbers of children for years to come. Furthermore, the scale-up of ART has reduced HIV-associated mortality dramatically so that CWH, who would previously have died in infancy or early childhood, are now reaching adolescence in increasing numbers. It is therefore important to understand the impact of HIV infection and its treatment on skeletal development during the critical period of puberty.

This study will determine the prevalence of low size-adjusted BMD in children with and without HIV in Zimbabwe, a country with a severe sustained early onset HIV epidemic. In addition, this study will

For peer review only - http://bmjopen.bmj.com/site/about/guidelines.xhtml

determine risk factors for low size-adjusted BMD in CWH. We aim to identify factors amenable to intervention, which may be modifiable to maximize future bone health and minimize subsequent adult osteoporotic fracture risk. For example, reduced muscle function predicting low size-adjusted BMD, may suggest targeted physiotherapy would be of benefit which would warrant formal investigation.

Our study will provide insights regarding the mechanisms through which perinatal HIV infection affects the timing of pubertal onset and bone mass accrual. By measuring bone and muscle parameters at baseline and one year and employing 'gold standard' size-adjustment methodology for DXA-measured BMD in the growing skeleton, this study will also provide insights into whether catch-up growth in terms of bone mass accrual is possible in HIV despite pubertal delay and provide age-related growth velocity data for CWH, with and without puberty.

Whilst the age range in this study, will allow analysis of pubertal delay in CWH, the follow-up period is insufficient to determine the impact on attainment of peak bone mass, which probably occurs in the early twenties [8]. In addition, this study is not sufficiently powered to analyse the effect of individual ART types on size-adjusted bone density. An additional limitation is the inability to obtain accurate height data for CWH prior to enrolment to fully study growth recovery. This is problematic given the significant role of poverty and nutrition, independent of HIV status, in the first 1000 days of childhood [53]; this may explain some of the deficit in final height attained by CWH.

The bone architecture measured by pQCT in this study will provide separate assessments of trabecular and cortical bone density, and bone geometry and strength in Zimbabwean children. The evidence from studies in adult men established on ART demonstrates impairments in trabecular and cortical bone architecture [54]. Whether the same applies to children needs to be determined.

Furthermore, we will establish novel comparator data for DXA, pQCT, bone age, hand grip strength and standing long jump for a Zimbabwean population, which will be able to be used for future research in this context. Although this represents the first steps towards developing normative reference data, the extent to which the children without HIV infection in this study are representative of the Zimbabwean population of 8 to 16 year olds is unknown. Furthermore, this study will establish a biorepository for future research *e.g.* potential bone turnover marker measurement and genotyping.

Given the magnitude of the HIV epidemic in SSA and the large cohort of young people who may experience impaired bone accrual, musculoskeletal disability or fracture as they reach adolescence

For peer review only - http://bmjopen.bmj.com/site/about/guidelines.xhtml

and early adulthood; it is imperative to characterise the impact of perinatal HIV on musculoskeletal development.

ETHICS AND DISSEMINATION

Ethical approval has been granted by the London School of Hygiene and Tropical Medicine Ethics Committee (Ref: 15333; 14 May 2018), the Institutional Review Board of the Biomedical Research and Training Institute (Ref: AP 145/2018; 20 February 2018), the Joint Research Ethics Committee for University of Zimbabwe College of Health Sciences and the Parirenyatwa Group of Hospitals (JREC) (Ref: 11/18; 1 March 2018), Harare Central Hospital Ethics Committee (HCHEC) (Ref: 170118/04; 23 February 2018), the Medical Research Council of Zimbabwe Ref: (MRCZ/A/2297; 10 April 2018) and the Ministry of Primary and Secondary Education Zimbabwe (Ref: C/426/Harare; 13 February 2018). This study is registered with the ISRCTN registry (Ref: ISRCTN12266984).

Study progress will be reported annually to MRCZ. Results of interim data analysis will be presented at national and international research meetings and conferences. Study findings will be published in international peer reviewed scientific journals and disseminated to research communities at the end of study.

AUTHORS' CONTRIBUTIONS

RR, RAF and CG co-designed the study. RR wrote the study protocol and was responsible for journal selection and preparation of the first draft of this article as the principal author. CK contributed to the development of the pQCT protocols. FK contributed to the development of the bone age analysis protocols. KW provided scan protocols, contributed to the study design, and gave methodological input regarding bone density size-adjustment and analysis. AR contributed to the study design, in particular, sampling strategy, sample size calculation and the statistical analysis plan. SF provided advice regarding the development of nutritional assessment tools. GM, SM and HM advised on study conduct and provided study oversight. All authors reviewed and provided feedback on the manuscript prior to submission.

FUNDING STATEMENT

This study is funded by the Wellcome Trust UK. RR is funded by Wellcome Trust UK grant number 206764/Z/17/Z. CK is funded by a National Institute of Health (NIH) Fogarty Trent Fellowship (Grant number 2D43TW009539-06). RAF is funded by Wellcome Trust grant number 206316/Z/17/Z. Global challenges research funding from the University of Bristol established the Sub-Saharan African MuSculOskeletal Network (SAMSON) enabling the provision of pQCT in Zimbabwe for this study. AMR

For peer review only - http://bmjopen.bmj.com/site/about/guidelines.xhtml

is additionally supported by the UK Medical Research Council (MRC) and the UK Department for International Development (DFID) under the MRC/DFID Concordat agreement which is also part of the EDCTP2 programme supported by the European Union grant reference (MR/R010161/1).

COMPETING INTERESTS STATEMENT

The authors have no competing interests to declare.

REFERENCES

1. UNICEF: **Monitoring the Situation of Children and Women; Global and regional trends, current status and progress.** https://datauniceforg/topic/hivaids/global-regional-trends/# 2017.
2. Celletti F, Sherman G, Mazanderani AH: **Early infant diagnosis of HIV: review of current and innovative practices**. *Curr Opin HIV AIDS* 2017, **12**(2):112-116.
3. Lowenthal ED, Bakeera-Kitaka S, Marukutira T, Chapman J, Goldrath K, Ferrand RA: **Perinatally acquired HIV infection in adolescents from sub-Saharan Africa: a review of emerging challenges**. *Lancet Infect Dis* 2014, **14**(7):627-639.
4. McGrath CJ, Chung MH, Richardson BA, Benki-Nugent S, Warui D, John-Stewart GC: **Younger age at HAART initiation is associated with more rapid growth reconstitution**. *AIDS* 2011, **25**(3):345-355.
5. WHO: **Growth failure in HIV-infected children**. In: *Consultation on Nutrition and HIV/AIDS in Africa: Evidence, lessons and recommendations for action.* Edited by SM A. Geneva, Switzerland: World Health Organisation, Department of Nutrition for Health and Development; 2005.
6. Arpadi SM, Shiau S, Marx-Arpadi C, Yin MT: **Bone health in HIV-infected children, adolescents and young adults: a systematic review**. *J AIDS Clin Res* 2014, **5**(11).
7. Compston J E: **Osteoporosis Review**. *Clinical Endocrinology* 1990, **33**(5):653-682.
8. Negredo E, Domingo P, Ferrer E, Estrada V, Curran A, Navarro A, Isernia V, Rosales J, Perez-Alvarez N, Puig J *et al*: **Peak bone mass in young HIV-infected patients compared with healthy controls**. *J Acquir Immune Defic Syndr* 2014, **65**(2):207-212.
9. Hernandez CJ, Beaupré GS, Carter DR: **A theoretical analysis of the relative influences of peak BMD, age-related bone loss and menopause on the development of osteoporosis**. *Osteoporos Int* 2003, **14**(10):843-847.
10. DiMeglio LA, Wang J, Siberry GK, Miller TL, Geffner ME, Hazra R, Borkowsky W, Chen JS, Dooley L, Patel K *et al*: **Bone mineral density in children and adolescents with perinatal HIV infection**. *AIDS* 2013, **27**(2):211-220.
11. Schtscherbyna A, Pinheiro MF, Mendonca LM, Gouveia C, Luiz RR, Machado ES, Farias ML: **Factors associated with low bone mineral density in a Brazilian cohort of vertically HIV-infected adolescents**. *International Journal of Infectious Diseases* 2012, **16**(12):e872-878.
12. Puthanakit T, Saksawad R, Bunupuradah T, Wittawatmongkol O, Chuanjaroen T, Ubolyam S, Chaiwatanarat T, Nakavachara P, Maleesatharn A, Chokephaibulkit K: **Prevalence and risk factors of low bone mineral density among perinatally HIV-infected Thai adolescents receiving antiretroviral therapy**. *J Acquir Immune Defic Syndr* 2012, **61**(4):477-483.
13. Matovu FK, Wattanachanya L, Beksinska M, Pettifor JM, Ruxrungtham K: **Bone health and HIV in resource-limited settings: a scoping review**. *Curr Opin HIV AIDS* 2016, **11**(3):306-325.
14. Slogrove AL, Schomaker M, Davies MA, Williams P, Balkan S, Ben-Farhat J, Calles N, Chokephaibulkit K, Duff C, Eboua TF *et al*: **The epidemiology of adolescents living with perinatally acquired HIV: A cross-region global cohort analysis**. *PLoS Med* 2018, **15**(3):e1002514.

For peer review only - http://bmjopen.bmj.com/site/about/guidelines.xhtml

15. Casado JL, Bañon S, Andrés R, Perez-Elías MJ, Moreno A, Moreno S: **Prevalence of causes of secondary osteoporosis and contribution to lower bone mineral density in HIV-infected patients**. *Osteoporosis International* 2014, **25**(3):1071-1079.

16. Weitzmann MN: **The Role of Inflammatory Cytokines, the RANKL/OPG Axis, and the Immunoskeletal Interface in Physiological Bone Turnover and Osteoporosis**. *Scientifica (Cairo)* 2013, **2013**:125705.

17. Aurpibul L, Cressey TR, Sricharoenchai S, Wittawatmongkol O, Sirisanthana V, Phongsamart W, Sudjaritruk T, Chokephaibulkit K: **Efficacy, safety and pharmacokinetics of tenofovir disoproxil fumarate in virologic-suppressed HIV-infected children using weight-band dosing.[Erratum appears in Pediatr Infect Dis J. 2015 Aug;34(8):847]**. *Pediatric Infectious Disease Journal* 2015, **34**(4):392-397.

18. Grant PM, Cotter AG: **Tenofovir and bone health**. *Current opinion in HIV and AIDS* 2016, **11**(3):326-332.

19. Hansen AB, Obel N, Nielsen H, Pedersen C, Gerstoft J: **Bone mineral density changes in protease inhibitor-sparing vs. nucleoside reverse transcriptase inhibitor-sparing highly active antiretroviral therapy: data from a randomized trial**. *HIV Med* 2011, **12**(3):157-165.

20. McComsey GA, Tebas P, Shane E, Yin MT, Overton ET, Huang JS, Aldrovandi GM, Cardoso SW, Santana JL, Brown TT: **Bone disease in HIV infection: a practical review and recommendations for HIV care providers**. *Clin Infect Dis* 2010, **51**(8):937-946.

21. Sudjaritruk T, Bunupuradah T, Aurpibul L, Kosalaraksa P, Kurniati N, Sophonphan J, Ananworanich J, Puthanakit T, Bone Dsg: **Impact of tenofovir disoproxil fumarate on bone metabolism and bone mass among perinatally HIV-infected Asian adolescents**. *Antiviral Therapy* 2016, **27**:27.

22. Mora S, Maruca K, Ambrosi A, Puzzovio M, Erba P, Nannini P, Benincaso A, Capelli S, Giacomet V: **Bone density, HIV infection and antiretroviral treatment: A 10-year follow-up in young patients**. *Hormone Research in Paediatrics* 2015, **84**:163-164.

23. Purdy JB, Gafni RI, Reynolds JC, Zeichner S, Hazra R: **Decreased bone mineral density with off-label use of tenofovir in children and adolescents infected with human immunodeficiency virus**. *J Pediatr* 2008, **152**(4):582-584.

24. Vancampfort D, Stubbs B, Mugisha J: **Physical activity and HIV in sub-Saharan Africa: a systematic review of correlates and levels**. *African health sciences* 2018, **18**(2):394-406.

25. Santos L, Elliott-Sale KJ, Sale C: **Exercise and bone health across the lifespan**. *Biogerontology* 2017, **18**(6):931-946.

26. Santos WR, Santos WR, Paes PP, Ferreira-Silva IA, Santos AP, Vercese N, Machado DR, de Paula FJ, Donadi EA, Navarro AM *et al*: **Impact of Strength Training on Bone Mineral Density in Patients Infected With HIV Exhibiting Lipodystrophy**. *J Strength Cond Res* 2015, **29**(12):3466-3471.

27. Dodds RM, Syddall HE, Cooper R, Kuh D, Cooper C, Sayer AA: **Global variation in grip strength: a systematic review and meta-analysis of normative data**. *Age Ageing* 2016, **45**(2):209-216.

28. Orsso CE, Tibaes JRB, Oliveira CLP, Rubin DA, Field CJ, Heymsfield SB, Prado CM, Haqq AM: **Low muscle mass and strength in pediatrics patients: Why should we care?** *Clinical Nutrition* 2019.

29. Macdonald E, Nettlefold L, Maan EJ, Cote H, Alimenti A: **Muscle power in children, youth and young adults who acquired HIV perinatally**. *J Musculoskelet Neuronal Interact* 2017, **17**(2):27-37.

30. Williams PL, Abzug MJ, Jacobson DL, Wang J, Van Dyke RB, Hazra R, Patel K, Dimeglio LA, McFarland EJ, Silio M *et al*: **Pubertal onset in children with perinatal HIV infection in the era of combination antiretroviral treatment**. *AIDS* 2013, **27**(12):1959-1970.

31. Szubert AJ, Musiime V, Bwakura-Dangarembizi M, Nahirya-Ntege P, Kekitiinwa A, Gibb DM, Nathoo K, Prendergast AJ, Walker AS, Team AT: **Pubertal development in HIV-infected African children on first-line antiretroviral therapy**. *AIDS (London, England)* 2015, **29**(5):609-618.

For peer review only - http://bmjopen.bmj.com/site/about/guidelines.xhtml

32. Kindblom JM, Lorentzon M, Norjavaara E, Hellqvist A, Nilsson S, Mellstrom D, Ohlsson C: **Pubertal timing predicts previous fractures and BMD in young adult men: the GOOD study**. *J Bone Miner Res* 2006, **21**(5):790-795.
33. Cousminer DL, Mitchell JA, Chesi A, Roy SM, Kalkwarf HJ, Lappe JM, Gilsanz V, Oberfield SE, Shepherd JA, Kelly A *et al*: **Genetically Determined Later Puberty Impacts Lowered Bone Mineral Density in Childhood and Adulthood**. *J Bone Miner Res* 2018, **33**(3):430-436.
34. Creo AL, Schwenk WF, 2nd: **Bone Age: A Handy Tool for Pediatric Providers**. *Pediatrics* 2017, **140**(6).
35. Crabtree N, Ward K: **Bone Densitometry: Current Status and Future Perspective**. In: *Calcium and Bone Disorders in Children and Adolescents. Volume Vol 28* 2nd, revised edition. edn. Edited by Allgrove J, Shaw NJ. Basel: Karger; 2015: pp 72-83.
36. Crabtree NJ, Shaw NJ, Bishop NJ, Adams JE, Mughal MZ, Arundel P, Fewtrell MS, Ahmed SF, Treadgold LA, Hogler W *et al*: **Amalgamated Reference Data for Size-Adjusted Bone Densitometry Measurements in 3598 Children and Young Adults-the ALPHABET Study**. *J Bone Miner Res* 2017, **32**(1):172-180.
37. Dennison EM, Jameson KA, Edwards MH, Denison HJ, Aihie Sayer A, Cooper C: **Peripheral quantitative computed tomography measures are associated with adult fracture risk: The Hertfordshire Cohort Study**. *Bone* 2014, **64**:13-17.
38. Stagi S, Cavalli L, Cavalli T, de Martino M, Brandi ML: **Peripheral quantitative computed tomography (pQCT) for the assessment of bone strength in most of bone affecting conditions in developmental age: a review**. *Italian journal of pediatrics* 2016, **42**(1):88-88.
39. Yin MT, Lund E, Shah J, Zhang CA, Foca M, Neu N, Nishiyama KK, Zhou B, Guo XE, Nelson J *et al*: **Lower peak bone mass and abnormal trabecular and cortical microarchitecture in young men infected with HIV early in life**. *AIDS* 2014, **28**(3):345-353.
40. **Parirenyatwa Group of Hospitals: https://parihosp.org**
41. Harare Central Hospital: **http://www.hararehospital.gov.zw**. 2019.
42. Government of Zimbabwe: **Harare Provincial Profile**. In. Harare: Parliament; 2011.
43. Rukuni R, McHugh G, Majonga E, Kranzer K, Mujuru H, Munyati S, Nathoo K, Gregson CL, Kuper H, Ferrand RA: **Disability, social functioning and school inclusion among older children and adolescents living with HIV in Zimbabwe**. *Tropical Medicine and International Health* 2017.
44. Simms V, Dauya E, Dakshina S, Bandason T, McHugh G, Munyati S, Chonzi P, Kranzer K, Ncube G, Masimirembwa C *et al*: **Community burden of undiagnosed HIV infection among adolescents in Zimbabwe following primary healthcare-based provider-initiated HIV testing and counselling: A cross-sectional survey**. *PLOS Medicine* 2017, **14**(7):e1002360.
45. Craig CL, Marshall AL, Sjöström M, Bauman AE, Booth ML, Ainsworth BE, Pratt M, Ekelund U, Yngve A, Sallis JF *et al*: **International physical activity questionnaire: 12-country reliability and validity**. *Med Sci Sports Exerc* 2003, **35**(8):1381-1395.
46. Filteau S, Rehman AM, Yousafzai A, Chugh R, Kaur M, Sachdev HPS, Trilok-Kumar G: **Associations of vitamin D status, bone health and anthropometry, with gross motor development and performance of school-aged Indian children who were born at term with low birth weight**. *BMJ Open* 2016, **6**(1).
47. FANTA: **Developing and Validating Simple Indicators of Dietary Quality and Energy Intake of Infants and Young Children in Developing Countries: Summary of findings from analysis of 10 data sets**. *Food and Nutrition Technical Assistance Project - Working Group on Infant and Young Child Feeding Indicators* 2006.
48. Foster HE, Jandial S: **pGALS - paediatric Gait Arms Legs and Spine: a simple examination of the musculoskeletal system**. *Pediatr Rheumatol Online J* 2013, **11**(1):44.
49. Crespi CM, Alfonso VH, Whaley SE, Wang MC: **Validity of child anthropometric measurements in the Special Supplemental Nutrition Program for Women, Infants, and Children**. *Pediatric research* 2012, **71**(3):286-292.
50. Baird J WI, Smith C, Inskip H. : **Review of methods for determining pubertal status and age of onset of puberty in cohort and longitudinal studies**. In: *Review of methods for*

For peer review only - http://bmjopen.bmj.com/site/about/guidelines.xhtml

determining pubertal status and age of onset of puberty in cohort and longitudinal studies. Edited by CLOSER. London, UK: CLOSER: MRC Lifecourse Epidemiology Unit, University of Southampton; 2017.

51. Abitbol L, Zborovski S, Palmert MR: **Evaluation of delayed puberty: what diagnostic tests should be performed in the seemingly otherwise well adolescent?** *Archives of Disease in Childhood* 2016, **101**:767-771.
52. Martin DD, Wit JM, Hochberg Z, Sävendahl L, van Rijn RR, Fricke O, Cameron N, Caliebe J, Hertel T, Kiepe D *et al*: **The Use of Bone Age in Clinical Practice – Part 1**. *Hormone Research in Paediatrics* 2011, **76**(1):1-9.
53. Schwarzenberg SJ, Georgieff MK: **Advocacy for Improving Nutrition in the First 1000 Days to Support Childhood Development and Adult Health**. *Pediatrics* 2018, **141**(2).
54. Biver E, Calmy A, Delhumeau C, Durosier C, Zawadynski S, Rizzoli R: **Microstructural alterations of trabecular and cortical bone in long-term HIV-infected elderly men on successful antiretroviral therapy**. *AIDS* 2014, **28**(16):2417-2427.
55. Clark EM, Ness AR, Tobias JH: **Bone fragility contributes to the risk of fracture in children, even after moderate and severe trauma**. *J Bone Miner Res* 2008, **23**(2):173-179.
56. Washington Group on Disability Statistics, UNICEF: **Module on Child Functioning and Disability** *Available online from http://wwwwashingtongroup-disabilitycom/wp-content/uploads/2016/02/wg_unicef_child-disability-background-documentpdf* 2014.
57. Marshall WA, Tanner JM: **Variations in pattern of pubertal changes in girls**. *Arch Dis Child* 1969, **44**(235):291-303.
58. Marshall WA, Tanner JM: **Variations in the pattern of pubertal changes in boys**. *Arch Dis Child* 1970, **45**(239):13-23.
59. **The WHO child growth standards.Growth reference, 5–19y.** [Geneva, Switzerland: World HealthOrganization; 2007 http://www.who.int/childgrowthref/en/13]
60. Häger-Ross C, Rösblad B: **Norms for grip strength in children aged 4-16 years**. *Acta Paediatr* 2002, **91**(6):617-625.
61. Armstrong M: **Youth Fitness Testing in South African Primary School Children: National Normative Data, Fitness and Fatness, and Effects of Socioeconomic Status**. Cape Town: University of Cape Town; 2009.

For peer review only - http://bmjopen.bmj.com/site/about/guidelines.xhtml

TABLES AND FIGURES

Table 1. Summary of study measurements to be quantified at baseline and follow-up

	Measurement	Measurement method	Outcome
INTERVIEW BASED QUESTIONNAIRE	Socio-demographic characteristics	Questionnaire	Age, sex, school attendance, orphanhood, guardianship
	Clinical history	Questionnaire[a]	History of fractures and trauma (modified Landin classification [55])<br>*HIV history: age at diagnosis, WHO disease stage, nadir CD4 count, opportunistic infections<br>*ART regimen/duration,<br>Exposures: steroid use, smoking, alcohol, recreational drugs<br>Family history of musculoskeletal disease & fractures<br>Other co-morbidities
	Physical activity	The International Physical Activity Questionnaire (IPAQ) [45] questionnaire<br>(short form)	Median MET-minutes[b] of physical activity/week<br>1. inactive (<600 MET-minutes/week)<br>2. minimally active (600-1499 MET-minutes/week)<br>3. highly active (≥1500 MET-minutes/week)
	Nutrition[b]	Dietary assessment tool (Modified short food frequency questionnaire [46])	Daily dietary calcium and vitamin D intake<br>Prevalence of vitamin supplementation<br>Sun exposure
	Quality of life and disability	Washington Disability Score [56]	Functioning and disability score
STANDARDISED EXAMINATION	Musculoskeletal examination	Paediatric Gait Arms Legs and Spine (pGALS)[48]<br>+/- regional clinical examination	Joint, spine and gait abnormalities
	Pubertal stage	Tanner's staging [57, 58]	Pre-pubertal (Stage 1)<br>Pubertal (Stage 2-3)<br>Post-pubertal (Stage 4 & 5)
	Anthropometry	Height (standing & sitting)<br>Weight<br>Mid-upper arm circumference (MUAC)[c]	Standing height-for-age (Z-score) [59][d]<br>Weight-for-age (Z-score) [59][d]<br>Body Mass Index (BMI) (Z-score) [59][d]<br>MUAC (Z-score) [59][d]
	Muscle strength	Jamar Dynamometer<br>Standing long jump[d]	Hand grip strength (kg, Z-score) [60][d]<br>Jumping distance (cm, Z-score) [61][e]
RADIOLOGY	Skeletal maturity	Hand/ wrist radiograph	Bone age (years)
	Bone and muscle composition	Dual-energy X-ray absorptiometry (DXA) of total body, lumbar spine and hip	Size corrected DXA measures of TBLH BMC^{LBM} (g), LS BMAD (g/cm^3) and Z-scores <-2.[d]<br>Lean mass
	Bone architecture	Peripheral quantitative computed tomography (pQCT)	Trabecular and cortical vBMD (g/cm^3),<br>Total and cortical CSA (mm^2), cortical thickness (mm), Periosteal and endosteal circumference (mm), SSI (mm^3) PMI (mm^4) and CSMI (mm^4)
BLOOD TESTS	Bone markers and DNA	Blood test (DNA extraction and serum saved)	Future testing
	HIV markers	Blood test	*CD4 count, HIV viral load

For peer review only - http://bmjopen.bmj.com/site/about/guidelines.xhtml

Table 1. Footnotes

a) Details of treatment and co-morbidities will be confirmed by patient-held medical records where available. **b)** Energy requirements defined in METS (multiples of the resting metabolic rate that give a score in MET-minutes). **c)** Nutritional indicator to include composite information from history (usual diet last month, sun exposure- vitamin D status) and clinical exam (MUAC). Similar methods have been used in other low income contexts [46]. **d)** Age and sex specific Z-scores for 1) *anthropometric measures:* will be determined using WHO child growth standards [59]; 2) hand *grip strength*: will be determined with reference to the uninfected comparison group and European normative data [60]; 3) *jumping distance:* will be determined using normative data from South Africa [61] 4) *low BMD* will be determined with reference to published paediatric Hologic DXA reference databases for LS BMAD and TBLH BMC^{LBM} Z-scores [36]. **e)** Standing long jump; the longest distance after two attempts will be recorded. **f)** Pregnancy urine dipstick in females prior to DXA if uncertain pregnancy status. **g)** Tests to be carried out on stored blood when further funding is secured.

*Denotes assessments to be carried out in HIV-infected participants only. **Abbreviations:** CSA (cross-sectional area), CSMI (cross sectional moment of inertia), LS BMAD (lumbar spine bone mineral apparent density) PMI (polar moment of inertia), SSI (Strength Strain Index), TBLH BMC^{LBM} (total-body less-head bone mineral content for lean mass adjusted for height).

Figure 1. Hypothesized changes in bone mass across the life-course in HIV-infected and uninfected individuals

Figure 2. Hypothesised growth scenarios to be assessed as interactions between pubertal stage and HIV status on change in bone mass

For peer review only

For peer review only - http://bmjopen.bmj.com/site/about/guidelines.xhtml

Figure 1. Hypothesized changes in bone mass across the life-course in HIV-infected and uninfected individuals

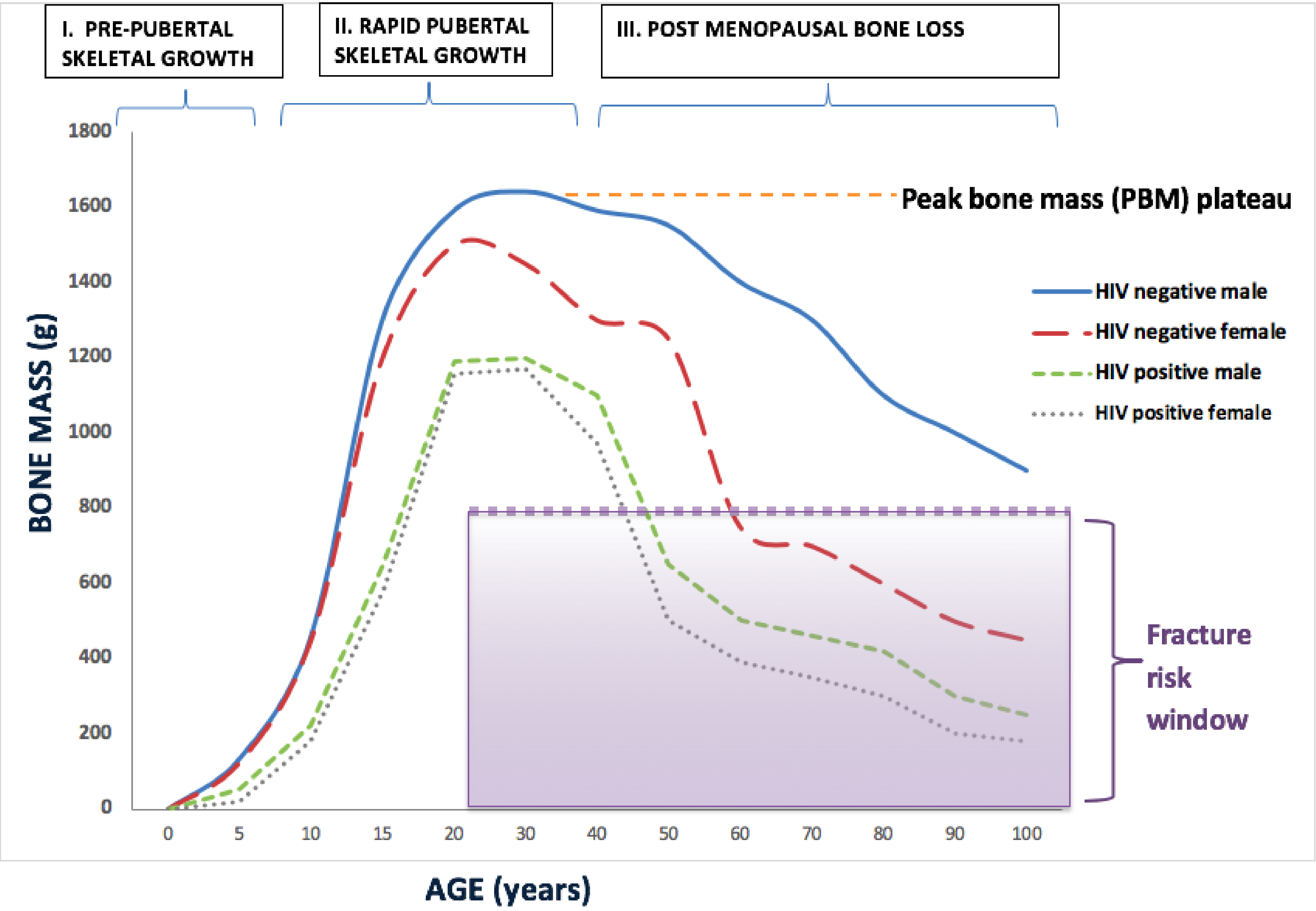

Figure 2. Hypothesised growth scenarios to be assessed as interactions between pubertal stage and HIV status on change in bone mass

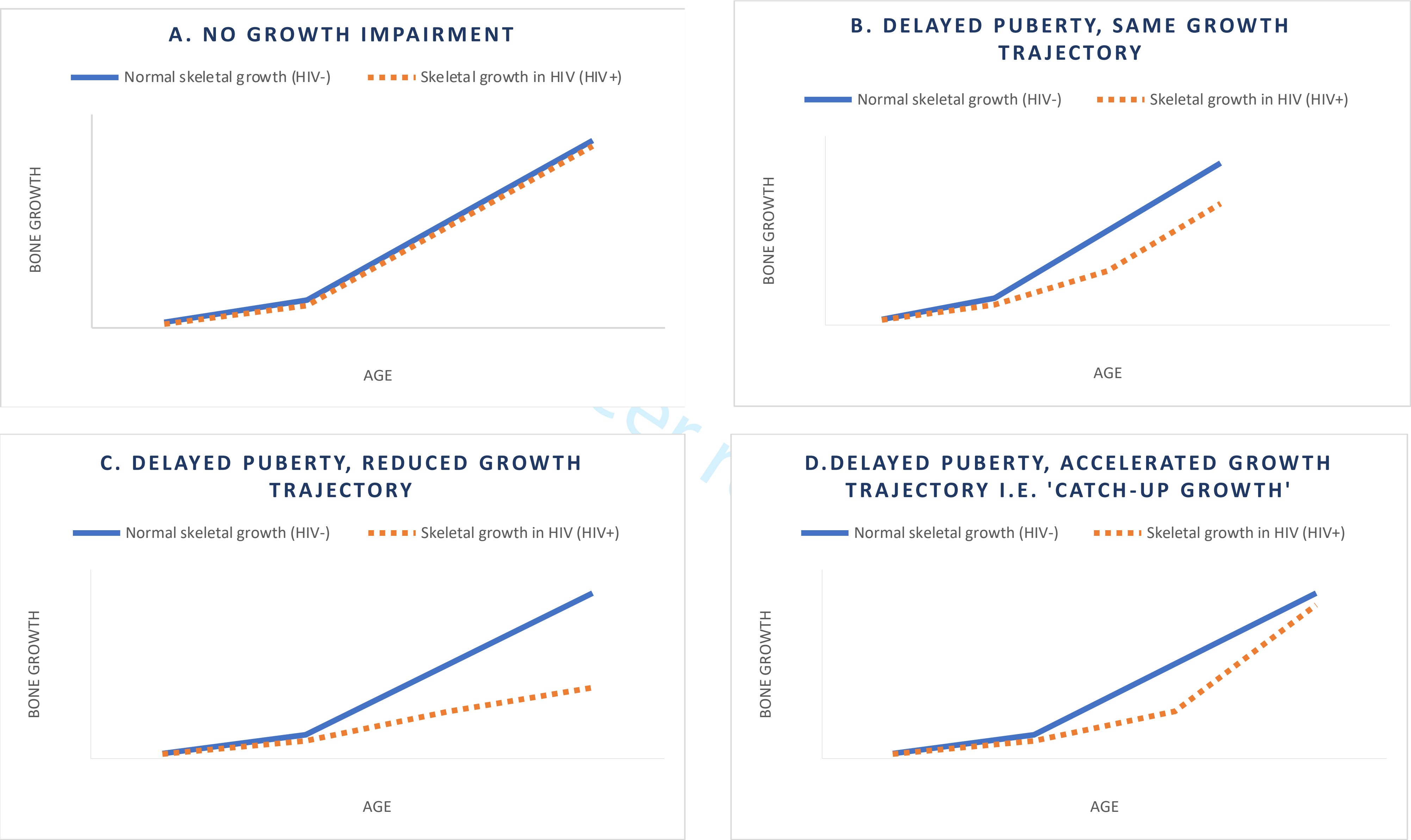

Supplement: Author's manuscript [file bmjopen-2019-031792.draft_revisions.pdf]
